# Unraveling the role of the CbrA histidine kinase in the signal transduction of the CbrAB two-component system in *Pseudomonas putida*

**DOI:** 10.1038/s41598-019-45554-9

**Published:** 2019-06-24

**Authors:** Elizabet Monteagudo-Cascales, Sofía M. García-Mauriño, Eduardo Santero, Inés Canosa

**Affiliations:** Universidad Pablo de Olavide, Centro Andaluz de Biología del Desarrollo/Consejo Superior de Investigaciones Científicas/Junta de Andalucía, Seville, Spain

**Keywords:** Bacteriology, Bacterial genetics

## Abstract

The histidine kinase CbrA of the CbrAB two-component system of *Pseudomonas putida* is a key element to recognise the activating signal and mediate auto- and trans-phosphorylation of the response element CbrB. CbrA is encoded by the gene *cbrA* which is located downstream of a putative open reading frame we have named *cbrX*. We describe the role of the CbrX product in the expression of CbrA and show there is translational coupling of the genes. We also explore the role of the transmembrane (TM) and PAS domains of CbrA in the signal recognition. A Δ*cbrXA* mutant lacking its TM domains is uncoupled in its growth in histidine and citrate as carbon sources, but its overexpression restores the ability to grow in such carbon sources. In these conditions ΔTM-CbrA is able to respond to carbon availability, thus suggesting an intracellular nature for the signal sensed.

## Introduction

Two-component systems (TCS) are one of the main mechanisms of signal transduction for bacteria to sense physicochemical and biological constraints to adjust their gene expression program and to respond to changing conditions properly. TCS regulate chemotaxis, sporulation, nutrient uptake and utilization among other important cellular processes^1,2^. The prototypical TCS consists of a membrane-bound sensor histidine kinase (HK) and a cytosolic response regulator (RR), which interacts with promoter regions to modulate DNA transcription. The input and output domains are divided into two separate proteins in a modular and versatile design and enables bacteria to respond not only to cytoplasmic stimuli but also to membrane-associated and extracellular signals^3,4^.

Signal transduction processes are typically initiated by the interaction of signal molecules with the sensor domains of the histidine kinases, which leads to an alteration of their autokinase activity and subsequently a change in the phosphorylation state of their RR partners, although a variety of alternative modes have evolved^3,4^. The enormous diversity of environmental signals and the fact that its perception has evolved through diverse mechanisms results in relatively few signals being identified in TCS systems^2,5^. Amongst the wide variety of sensor domains (more than 14 different types have been described in HKs), PAS domains are most frequently found. They are ubiquitously present in all kingdoms of life and sense a wide range of stimuli. PAS domains do not have conservation at the amino acid sequence level, thus hampering domain annotation, but usually adopt a conserved α/β-fold structure^6^. On the other hand, signal interaction with transmembrane regions has been reported for some temperature-sensing HKs^7^ or HK responding to antibiotics^8^. Finally, mechanisms have evolved in which the signal does not directly interact with the HK, but the stimulus is transmitted through an accessory protein. This mechanism is often called three-component system and the increasing number of reports suggests that such systems are more frequent than initially anticipated. The accessory proteins include methyl-accepting chemotaxis proteins (MCPs), a cytosolic accessory protein (i.e. PII for NtrB) or a periplasmic accessory protein (i.e. LuxP for LuxQ) (see^3^ and references therein).

In the *Pseudomonadaceae*, CbrA is a histidine kinase that functions as a global regulator of carbon metabolism, amino acids uptake, virulence or antibiotic resistance^9–15^. CbrA represents a new family of sensor HKs as its structure suggests it may link signalling to transport of a molecule. Its N terminus contains a 13 TM domain with similarity to the SLC5 transporter family and the sodium-proline symporter PutP from *E. coli*^16^, which is connected to the C terminal catalytic HK domain through a STAC (Solute carrier 5 (SLC)- and Two-component signal transduction Associated Component) motif, which could regulate transport through the sodium/solute symporter domain, based on structural and bioinformatics analyses^17,18^. Its cognate response regulator CbrB regulates expression of different σ^N^ -dependent metabolic genes^19–23^. It also participates in carbon catabolite repression control by the enhanced activity when growing in less-favored compounds. This leads to an increase in the abundance of the non-coding small RNA CrcZ (alone or together with CrcY in *P. putida*), that inhibit the activity of the Crc global regulator, along with Hfq, that act as translational repressors of its targets^22,24–26^.

In this work we present evidence on the role of CbrA in the signal transduction in the TCS CbrAB of *P. putida* and describe its transcriptional regulation. We also analyse the importance of the TM and PAS domains for the sensor function. Finally, our data on a CbrA mutant lacking its TM domains suggest an intracellular nature for the signal sensed by the system.

## Results

### A highly conserved *orf* upstream of *cbrA* was recently annotated in the *Pseudomonas* database

**Figure 1:**
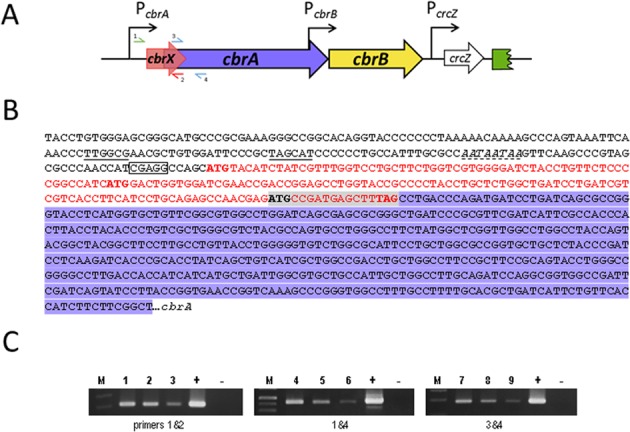
Genomic organisation of the *cbrX-cbrA-cbrB* cluster in *P. putida*. (**A**) Graphic representation of the gene organisation of *cbrX* (*PP5704* in red), *cbrA* (*PP4695* in purple), *cbrB* (*PP4696* in yellow) and *crcZ* (*PPmr53* in white) in *Pseudomonas putida* KT2442. The promoters for *cbrA*, *cbrB* and *crcZ* are depicted as black arrows. (**B**) DNA sequences for the promoter region and of *cbrX* and 5′ of *cbrA* in *Pseudomonas putida* KT2440. Underlined sequences correspond to the putative -10 and -35 boxes, a predicted Crc binding site is shown in italics and with a dotted underline, and the putative RBS for *cbrX* “CGAGG” is boxed. The coding sequence for *cbrX* is represented in red characters, that of *cbrA* is shaded in purple, and the overlapping sequences for *cbrX* and *cbrA* are shaded in grey. The start and stop codons are highlighted in bold. (**C**) *cbrX* and *cbrA* are co-transcribed in the same transcriptional unit. RT-PCR of 25, 5 and 1 ng of cDNA from a culture KT2442 growing in OAA was amplified with different combination of the primers represented in panel A; primers 1 (RT-PcbrXA_fwd2) and 2 (RT-cbrX_rev) (lines 1 to 3), primers 1 (RT-PcbrXA_fwd2) and 4 (RT-CbrA_rev) (lines 4 to 6) and primers 3 (RT-CbrA_fwd) and 4 (RT-CbrA_rev) (lines 7 to 9). Positive and negative PCR controls (+and −, respectively) were performed with genomic or no DNA for each pair of oligos (see Methods). M is the DNA molecular weight marker GeneRuler^TM^ 1 Kb Plus DNA ladder (ThermoFisher Scientific).

Recently, a new open reading frame (*PP5704*) that is upstream and overlapping *cbrA*, has been annotated in the *Pseudomonas* database (Pseudomonas.com) encoding a hypothetical protein (Fig. 1). Its nucleotide sequence overlaps the coding region of *cbrA* at its 3′ end by 17 nucleotides and is encoded in the second reading frame of *cbrA*. The mRNA detected by RT-PCR using two primers annealing at the leader untranslated region of *cbrXA* (primer 1 in Fig. 1A), and in the 3′ end of *cbrX* (primer 2) or within *cbrA* (primer 4) show that *cbrX* and *cbrA* are transcribed within the same transcriptional unit and there are no internal promoters that made a significant contribution to *cbrA* transcription independently of *cbrX* (Fig. 1C). There are also no major differences in the mRNA abundance compared to the products generated a combination of primers annealing within *cbrA* coding region (primers 3&4), thus outruling a major contribution of a possible promoter that would transcribe *cbrA* in a *cbrX* independent manner.

**Figure 2:**
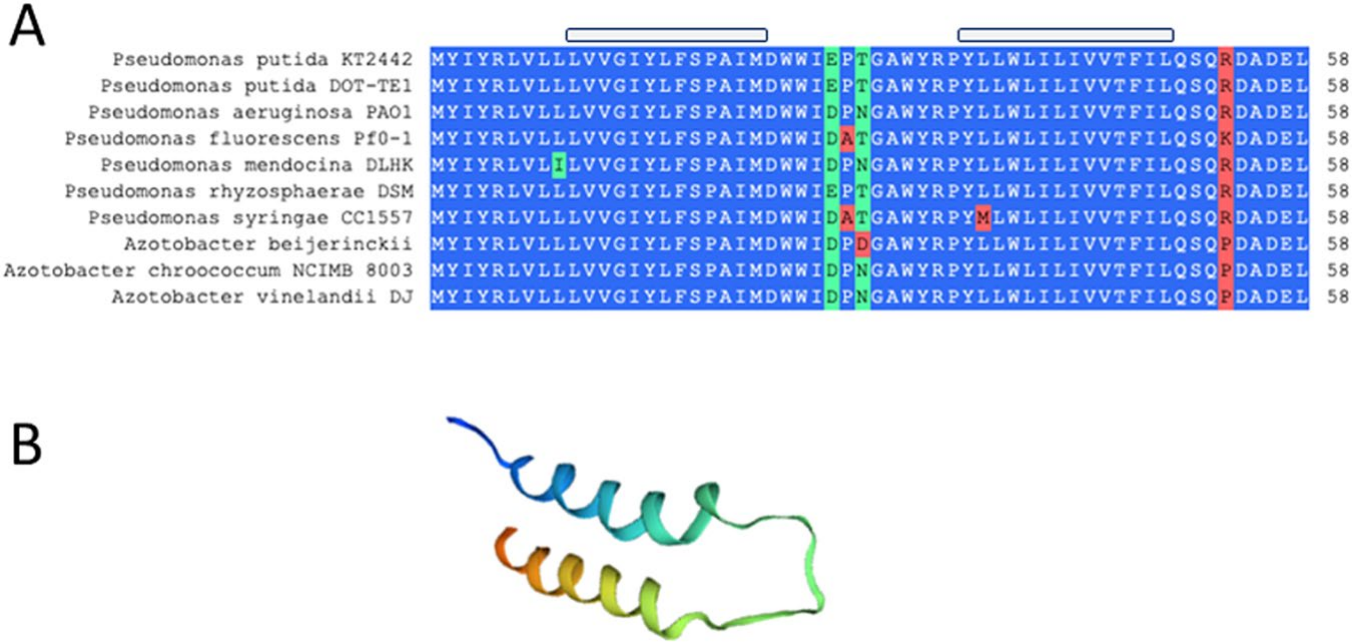
CbrX sequence conservation and predicted secondary structure (**A**) Sequence alignment on CbrX in different strains of the family *Pseudomonadaceae*. Relevant features are marked as follows: Blue aminoacids are identical, green are similar amino acids and red are non identical amino acids between strains. The span of the structural motifs shown in (**B**) are indicated on the top of the aligned sequences as a shaded box. (**B**) Predicted secondary structure of CbrX by SWISS-MODEL protein structure homology-modelling (ExPASy web server).

*cbrX* encodes a small 58 amino acids peptide of unknown function and is well conserved within the genera *Pseudomonas* and *Azotobacter* from the *Pseudomondaceae* family (Fig. 2A). It has no predicted paralogs in *Pseudomonas putida* and its secondary structure displays two α-helixes putatively inserted in the inner membrane of the cell (Fig. 2B), possibly close to the transmembrane domains of CbrA. To study the role of the CbrA domains and a possible effect of *cbrX* on *cbrA* expression, we constructed a deletion mutant derived from strain KT2442 that lacked the complete coding sequence for *cbrX* and *cbrA*, as well as their promoter region. The resulting Δ*cbrXA* deletion mutant, denoted MPO494, was confirmed by Southern blot after the double recombination event of a plasmid containing the flanking regions of the genes (see Methods and Fig. S1). Complementation of such strain with different versions of *cbrX* and/or *cbrA* by insertion of a miniTn7-directed fragment into the *glmS* locus of the chromosome, allowed to investigate the activity of the Cbr system in the presence or absence of CbrA and CbrX.

### The Δ*cbrXcbrA* null mutant shows the same phenotype as the Δ*cbrB* mutant

**Figure 3:**
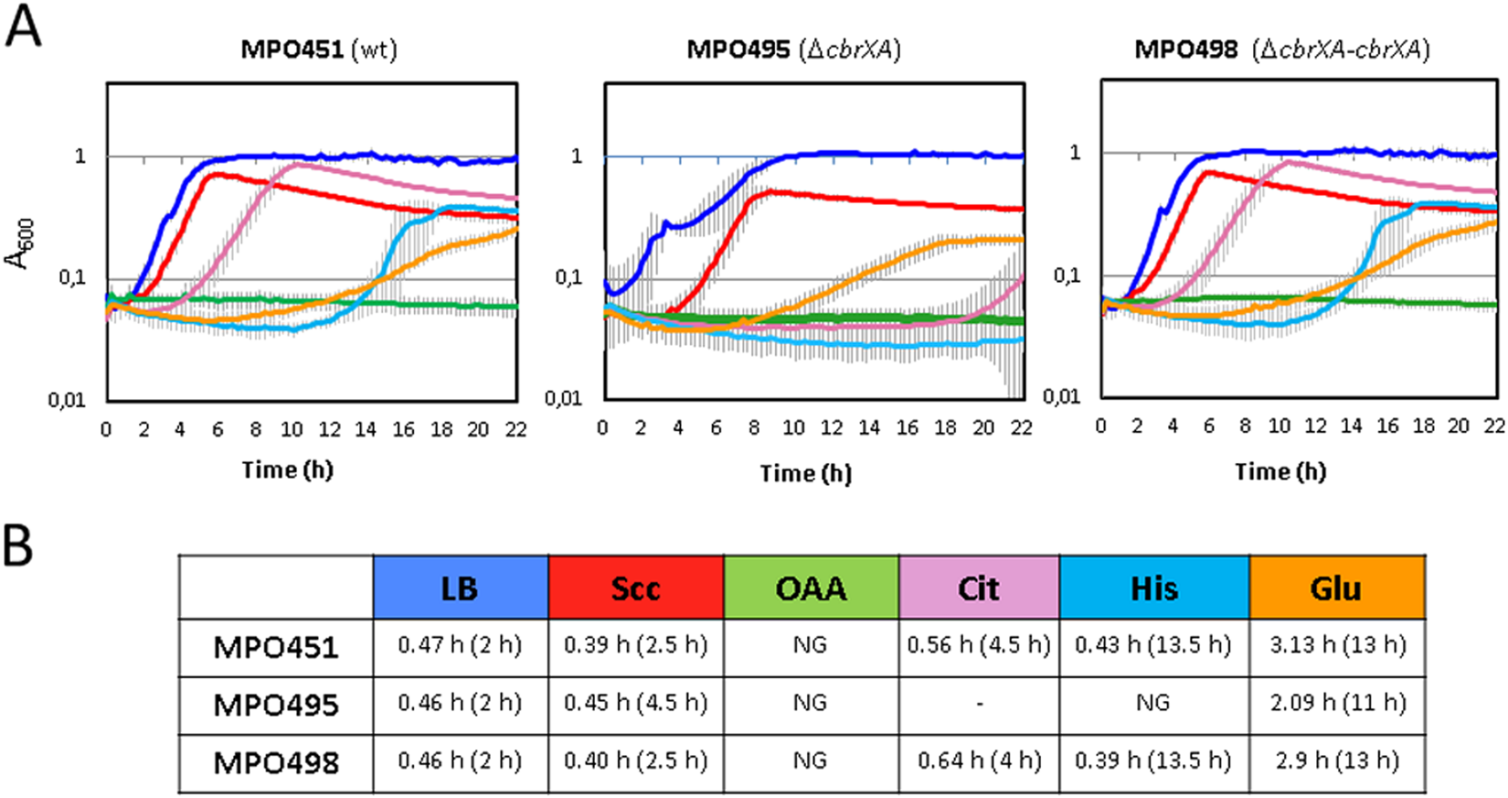
Growth of *P. putida* wild type, Δ*cbrXA* mutant and complemented strain in different carbon sources. (**A**) Growth curves of the wild type strain (MPO451), deletion mutant Δ*cbrXA* (MPO495) and complemented strain (MPO498) in LB and in minimal medium with succinate (Scc, red), oxalacetate (OAA, green), citrate (Cit, pink), histidine (His, cyan) and glucose (Glu, orange) as sole carbon sources. Three biological replicas have been performed for each growth curve but only one representative biological experiment is shown. Error bars represent the standard deviation of the three technical replicates of the same biological experiment. (**B**) Doubling time and lag phase of strains MPO451, MPO495 and MPO498 in rich medium (LB) and M9 medium with succinate (Scc), oxaloacetate (OAA), citrate (Cit), histidine (His) or glucose (Glu) as C sources. Lag phase in each condition is indicated in brackets. NG; no growth, (−); growth after 20 hours. Generation time was calculated during the exponential phase of growth as an extrapolation onto an exponential equation.

The ability of the wild type strain MPO451 (containing an empty miniTn7 from the plasmid pME6182 inserted in *glmS*) to grow in an LB rich and in a minimal media containing different compounds as carbon sources (succinate, OAA, citrate, histidine and glucose) was analysed together with that of the mutant Δ*cbrXA* (MPO495) and a mutant strain complemented with the complete *cbrX-cbrA* sequence contained within the miniTn7 (MPO498). The Δ*cbrXA* strain was not able to use citrate or histidine, but this ability was restored when the wild type sequence was reintroduced in the complemented strain MPO498 (Fig. 3A). The growth rate for the Δ*cbrXA* mutant strain in succinate was not significantly affected as compared to the wild type, but the lag phase increased by 2 hours, thus showing a longer time for adaptation to the new conditions (Fig. 3A,B). All three strains were unable to use OAA as carbon source within the timeframe of the experiment. It is interesting to highlight the fact that the *cbrXA* deletion mutant presents the same phenotypes as a deletion mutant on *cbrB* (MPO401) concerning the use of carbon sources^15,20^. The Δ*cbrXA* mutant is also affected in swimming motility in a similar extent as a Δ*cbrB* deletion strain, an it was restored upon complementation with a copy of *cbrXA* in *trans* (Fig. S2). It is worth mentioning that *cbrB* levels were not altered in the mutant strain Δ*cbrXA* since the P*cbrB* promoter was intentionally mantained unaltered at the 3′ end of *cbrA*. Nevertheless the actual *cbrB* mRNA levels were quantified by RT-qPCR and showed no significant variation in a KT2442 wild type and MPO494 *cbrXA* mutant strains in any condition of carbon availability (Fig. S3).

These data show that at least these processes require CbrA as the histidine kinase that triggers CbrB activation, and there is no direct crosstalk with any of the other 59 putative histidine kinases encoded in the genome of strain KT2442 in a CbrA independent manner (Pseudomonas.com).

**Figure 4:**
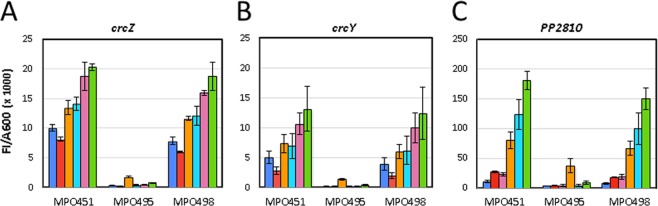
Expression levels of *crcZ, crcY* and *PP2810* in *P. putida* wild type, Δ*cbrXA* mutant and complemented strains on different carbon sources. The expression levels are represented as the fluorescence intensity (FI) detected from *gfp* normalised by the optical density at A_600_ for strains MPO451 (wild type), MPO495 (Δ*cbrXA*) and MPO498 (Δ*cbrXA-cbrXA*), bearing transcriptional fusions of *crcZ* (A)*, crcY* (B) or *PP2810* (**C**) promoters to *gfp* (in pMPO356, pMPO357 and pMPO355, respectively). The expression levels after 8 hours of induction are presented in a LB rich medium (blue), in a minimal medium containing succinate (red), glucose (orange), histidine (cyan), citrate (pink) and oxaloacetate (green). Y-axis scale is not comparable for the three panels. Panels A and B show the FI measured with a gain of 35 due to the *crcZ* and *crcY* high expression levels while panel C (PP2810) is measured at the default gain of 55. One representative experiment of the three biological replicates is represented, and error bars represent the standard deviation of three technical replicates for the biological replicate shown.

In addition, the CbrB activity was analysed as the transcriptional activation of three previously defined CbrB targets^23^, as the quantification of the fluorescence intensity of a *gfp-lacZ* transcriptional fusions to the promoter regions of *crcZ*, *crcY* and *PP2810* (plasmids pMPO356, pMPO357 and pMPO355, respectively). PP2810 is the third characterised CbrB direct target and codes for an efflux pump. It is included in this characterisation since it shows the lowest basal expression levels under repressing conditions (LB) and the highest induction rate in non-repressing conditions (OAA) of all CbrB targets^23^. Figure 4 shows the differential induction of the targets *crcZ*, *crcY* and *PP2810* in LB, and in a minimal medium containing succinate, glucose, histidine, citrate or oxaloacetate as carbon sources. In the wild type strain (MPO451), the three targets showed the same pattern of expression, and the maximal induction was detected in the conditions where non-preferential carbon sources were present. This data is completely coherent with the β-galactosidase activity of the same targets described previously^13^ showing that measurement of fluorescence intensity from *gfp* transcriptional fusions is linear and reproducible. The minimal medium containing OAA was the condition that showed the highest levels of activity for the three genes, followed by histidine, citrate and glucose at similar extent, and finally succinate as the most repressing source. The LB rich medium was as repressor as succinate, which is the source preferentially assimilated by *Pseudomonas*. Nevertheless, in the wild type strain the target *PP2810* showed the best ability to discriminate between carbon sources, and was selectively induced to a different extent depending on the carbon source present. In addition, it showed the lowest levels of expression in repressing sources such as LB or succinate, whereas *crcZ* and *crcY* were partially derepressed (Fig. 4).

Activation of the three targets was strictly dependent on CbrA in all media, as shown in a Δ*cbrXA* background (MPO495), and was fully recovered after complementation with a complete copy of the genes in the miniTn7 (MPO498). Only in the medium containing glucose as carbon source a CbrA-independent basal activity was detected for the three genes (Fig. 4).

### CbrA expression is dependent on carbon availability

**Figure 5:**
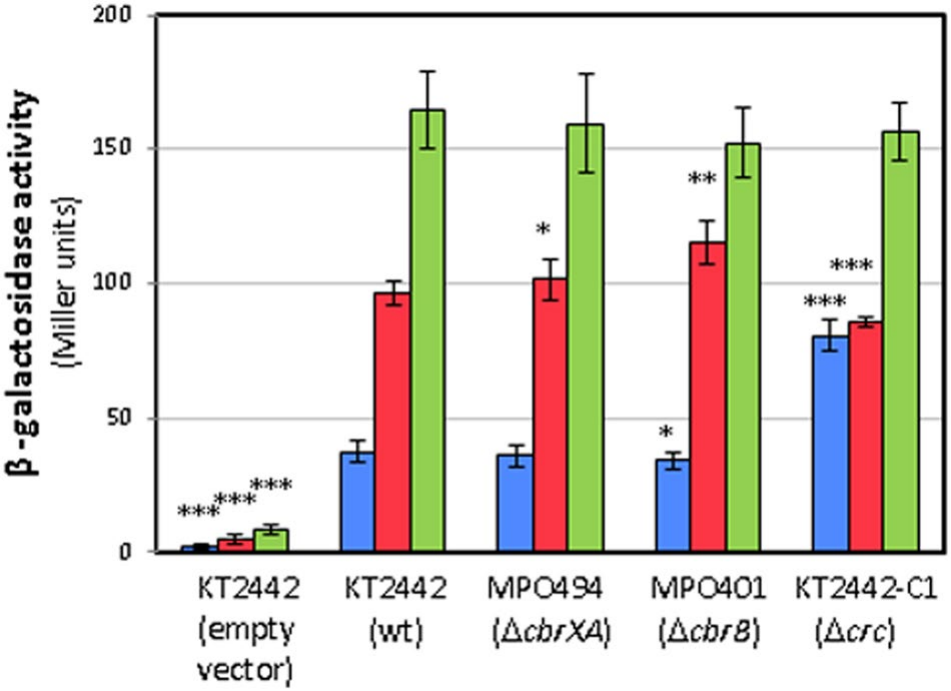
Expression levels of CbrA in a wild type, Δ*cbrXA*, Δ*cbrB* and Δ*crc* backgrounds. Expression was measured as β-galactosidase activity of a translational fusion of *cbrA*’-‘*lacZ* in plasmid pMPO1370 (coord. +249) for the wild type (KT2442), Δ*cbrXA* (MPO494), Δ*cbrB* (MPO401) and Δ*crc* (KT2442-C1) strains. The assays were performed in LB medium (blue), minimal medium with succinate (red) or oxaloacetate (green) as carbon sources. pMPO200 empty plasmid was used as control. The values are the average of at least three independent assays, and error bars indicate the standard deviation of the means. Stars designate p-values for the Student’s t-test for unpaired samples not assuming equal variance. *:p < 0.05; **p < 0.01; ***p < 0.005.

We quantified the expression of CbrA in conditions of variable carbon availability by β-galactosidase activity measurement of a translational fusion of *cbrXA* to *lacZ* in plasmid pMPO200 to produce pMPO1370. Maximal levels of CbrA were obtained for a KT2442 wild type strain in conditions of carbon limitation in a medium containing OAA as carbon source, with expression levels 2-fold higher than in succinate and 5-fold higher than in a rich LB medium (Fig. 5). Nevertheless, the expression was not dependent on CbrA since in a *cbrXA* background (MPO494) it reached the same levels as those in the wild type. The expression pattern was also conserved in a Δ*cbrB* background (MPO401), thus showing these regulated expression levels are also independent of the phosphorylation status of the response regulator. Finally, the dependence on Crc was also assayed and the Δ*crc* mutant showed a 2-fold increased expression in LB, but no significant effect on defined minimal medium regardless the carbon source (Fig. 5). This data shows that Crc is preventing *cbrA* expression in the conditions of its maximal activity, where the levels of the small RNAs CrcZ and CrcY are minimal and Crc is unrestricted for mRNA binding.

### CbrX is essential for CbrA production

**Figure 6:**
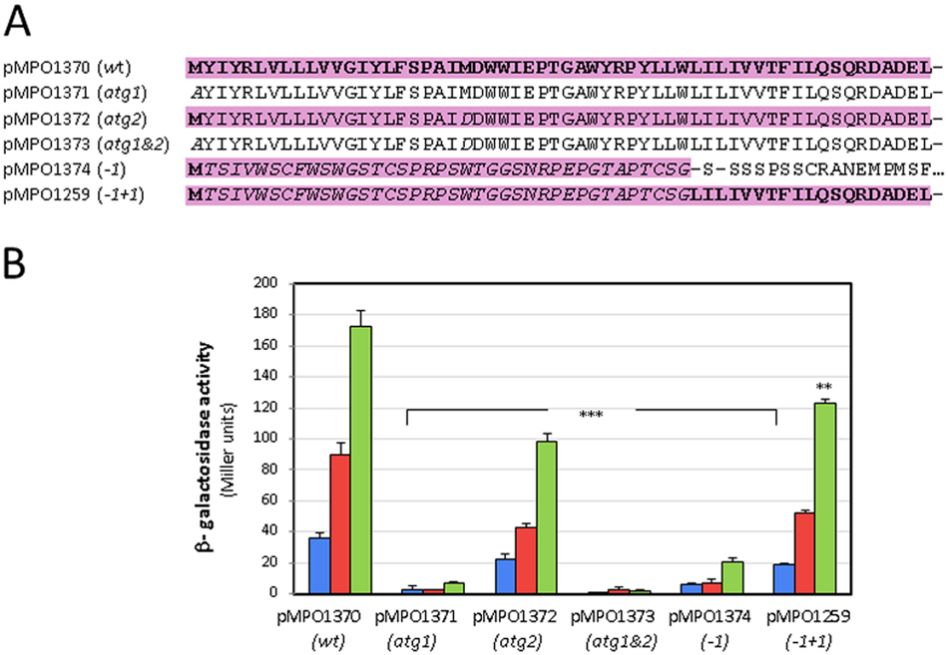
Point mutations in *cbrX* and their effect on CbrA expression. (**A**) The reading frame of CbrX for the wild type *cbrXA* sequence (pMPO1370; wt) and point mutations at the putative start ATG (pMPO1371, atg1), at an ATG codon downstream (pMPO1372; atg2), both ATG codons mutated (pMPO1373; atg1&2), a T deletion at coordinate +4 from the ATG of *cbrX* (pMPO1374; -1) and a T deletion and subsequent reversion of the reading frame with a C insertion at +118 from the ATG (pMPO1259; -1, +1) are represented. Residues corresponding to CbrX in its wild type reading frame are shown in bold, the predicted amino acid sequences of translated peptides are shaded in pink. Amino acids in italic are residues not coincident with the wild type CbrX sequence. (**B**) Quantification of *cbrA* expression by β-galactosidase activity of translational *cbrA*’-‘*lacZ* fusions carrying wild type sequence (pMPO1370) and point mutations in *cbrX* sequence in pMPO1371, pMPO1372, pMPO1373, pMPO1374, pMPO1259. β-galactosidase activity was monitored in *P. putida* KT2442 in LB (blue) and M9 medium supplemented with succinate (red) or oxalacetate (green). Bars represent the averages and standard deviations of at least three independent assays. Stars designate p-values for the Student’s t-test for unpaired samples not assuming equal variance. *p < 0.05; **p < 0.01; ***p < 0.005.

With the purpose to elucidate a possible effect for CbrX in the expression of CbrA, translational fusions *cbrXA*’-‘*lacZ* bearing the WT sequence or point mutations in *cbrX* were constructed in plasmids and transformed into the strain KT2442. The levels of CbrA were monitored by β-galactosidase activity assays. Point mutations in the coding region of *cbrX* were generated from the wild type sequence (see Methods). The DNA sequences containing all the *cbrX* mutations and the corresponding effect on CbrX translation are detailed in Fig. S4. The modifications included a point mutation of the first ATG (AUG to GCA) in plasmid pMPO1371, a point mutation in a second ATG codon located 60 bp downstream of the first ATG (AUG to GAU) in plasmid pMPO1372, a double mutant containing both ATG codons mutated in plasmid pMPO1373, a frameshifting deletion of the T at position +4 in plasmid pMPO1374, and the same deletion with a subsequent recovery of the reading frame at position +118 (insertion of a C) in plasmid pMPO1259. In this case, a peptide of the same length as CbrX is produced, thus maintaining the potential translational coupling between *cbrX* and *cbrA*, but with a different sequence (Fig. 6A). The quantification of β-galactosidase activity from the constructions bearing all these *cbrX* variants is shown in Fig. 6B, and showed accurately the amount of translated CbrA at each condition in the wild type background. The levels of CbrA differed depending on the carbon availability for the wild type *cbrX* sequence (plasmid pMPO1370) and increased in 2 and 5 -fold in OAA compared to succinate and LB, respectively. When the predicted *cbrX* initiation codon was mutated (pMPO1371; i.e. no CbrX produced), expression of CbrA was very low (Fig. 6B), thus indicating that translation of *cbrX* is important for *cbrA* translation. Mutation of the second ATG codon of *cbrX* (ATG2; in plasmid pMPO1372) located downstream resulted in a 1.7-fold decrease of CbrA expression though it retained significant expression in all conditions. Mutation of both ATGs resulted in similar or slightly lower levels of CbrA to those with the ATG1 mutation alone. Although the 1.7-fold reduction in CbrA expression levels caused by the ATG2 mutation in *cbrX* could suggest that the second ATG codon of *cbrX* might also be used as an initiation codon but with less efficiency, the fact that expression in the ATG1 mutant was reduced to levels similar to those of the empty vector (shown in Fig. 5) indicates that the second ATG could not be used as the initiation codon in the ATG1 mutant. Thus, the effect of the ATG2 mutation is probably a consequence of the alteration of the CbrX amino acid sequence and not to the use of an alternative initiation codon. Therefore, these results showed that ATG1 is the actual initiation codon for *cbrX* translation.

Altering the reading frame of *cbrX* by deleting a T after the ATG (at position +4 ; plasmid pMPO1374) should result in premature translation termination and production of a truncated peptide with a different sequence (see Fig. 6A). This mutation resulted in strong reduction of *cbrA* translation (Fig. 6B). Nevertheless, when the reading frame that was previously altered was recovered by the insertion of a C upstream from the translational start of CbrA (plasmid pMPO1259), a peptide with the same length and Ct end as that of the wild type CbrX, but different Nt sequence, was generated. Translation of this different peptide allowed production of CbrA to almost the same levels as those with the wild type CbrX (Fig. 6B). These results clearly suggest that it is the translation of the *cbrX* orf down to reach *cbrA* and not its identity as a peptide what is required for CbrA translation, which indicate that both genes are translationally coupled. To validate that the effect was due to reduced translation and not to a difference in mRNA levels, RT-PCR was performed with oligos hybridising to the *cbrX* and *cbrA* coding regions. These analyses showed that the relative mRNA levels of both orfs were similar in the wild type strain (MPO451) and in the mutants containing point mutations in the *cbrX* sequence (Fig. S5). All this data show that CbrA requires CbrX to be translated in a process of translational coupling. Additionally, alteration of the CbrX sequence in the −1 + 1 construct (Fig. 6A) yields a peptide with a different secondary structure that does not correspond to an α-helix by structure prediction algorithms, thus it is highly improbable that the function of CbrX is related to its identity as a peptide or its insertion in the membrane.

### Basal amounts of CbrA are sufficient for *crcZ* activation

**Figure 7:**
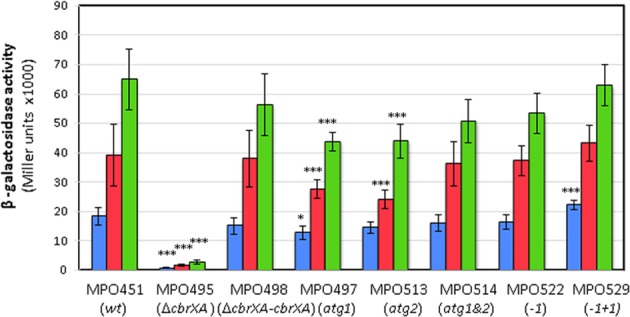
Expression levels of *crcZ* in a wild type and in strains with different variants of CbrX. β-galactosidase activity was measured for pMPO1316 containing a transcriptional fusion of *crcZ* to *lacZ* in MPO451 (wild type), MPO495 (Δ*cbrXA*), MPO498 (Δ*cbrXA-cbrXA*), MPO497 (Δ*cbrXA*-ATG1), MPO513 (Δ*cbrXA-*ATG2), MPO514 (Δ*cbrXA*-ATG1&2), MPO522 (Δ*cbrXA*-ΔT) and MPO529 (Δ*cbrXA*-ΔT + C). Activity was monitored in LB (blue) and minimal medium supplemented with succinate (red) or oxalacetate (green). Bars represent the averages and standard deviations of at least three independent assays. Stars designate p-values for the Student’s t-test for unpaired samples not assuming equal variance. *:p < 0.05; **p < 0.01; ***p < 0.005.

To evaluate the effect of CbrX in the CbrAB activation ability we complemented a Δ*cbrXA* mutant (MPO494) with *cbrXA* inserted into the chromosome with the miniTn7, containing the wild type *cbrX* and mutated versions assayed above. The strains containing the empty plasmid (MPO495), the wild type *cbrXA* (MPO498), mutation of ATG1 (MPO497), ATG2 (MPO513), ATG1 and ATG2 (MPO514), deletion of a T after ATG1 (MPO522) and restoration of the reading frame by a C insertion (MPO529) were generated. Also, the wild type KT2442 with the insertion of the empty plasmid was used as control (MPO451). All the strains were transformed with plasmid pMPO1316 containing a *crcZ::lacZ* transcriptional fusion. Measurement of the β-galactosidase activity revealed that complementation of the *cbrXA* sequence restored the *crcZ* transcription levels to the wild type levels as expected (MPO498 compared to MPO451 in Fig. 7). Surprisingly, the expression levels for *crcZ* were not obviously affected in strains MPO497, MPO513, MPO514 and MPO522, where the amount of CbrA produced was clearly lower (as shown in Fig. 6). This means that the low levels of CbrA produced in these *cbrX* mutants are enough for CbrB phosphorylation and consequent *crcZ* transcription activation.

### The PAS domain is essential for Cbr mediated signal transduction and the TM domains of CbrA are dispensable for signal detection

**Figure 8:**
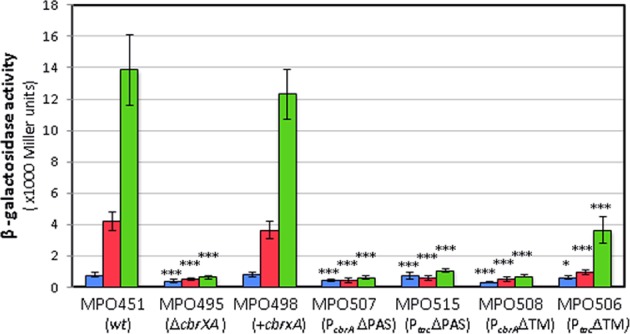
Expression levels of *PP2810* in strains with ΔTM and ΔPAS truncated variants of CbrA. β-galactosidase activity was measured for pMPO420 (*PP2810*::*lacZ*) in strains MPO451 (wild type), MPO495 (Δ*cbrXA*), MPO498 (Δ*cbrXA*-*cbrXA*), MPO507 (Δ*cbrXA-* PAS), MPO515 (Δ*cbrXA*-P_*tac*_ΔPAS), MPO508 (Δ*cbrXA*-ΔTM) and MPO506 (Δ*cbrXA-*P_*tac*_ΔTM). Bars represent the averages and standard deviations of at least three independent assays. Stars designate p-values for the Student’s t-test for unpaired samples not assuming equal variance. *p < 0.05; **p < 0.01; ***p < 0.005 comparing with MPO498 transcriptional levels.

CbrA contains several well conserved domains in all the species of *Pseudomonas*, including 13 transmembrane domains at the Nt followed by a PAS domain, which precedes the autokinase domain at the Ct. We constructed truncated CbrA versions lacking the TM or PAS domains, expressed from its own promoter or expressed from a P_*tac*_ strong promoter. All these constructions were inserted into the chromosome of the Δ*cbrXA* mutant strain MPO494 using miniTn7, and their effect on CbrB-mediated transcription activation of the PP2810 target using a *PP2810-lacZ* reporter fusion in plasmid pMPO420 was evaluated. The β-galactosidase activity of these constructs are shown in Fig. 8. The deletion of the PAS domain (ΔPAS) resulted in a CbrA product of 915 aminoacids that did not activate the *PP2810* target neither in its physiological conditions (MPO507) nor when overexpressed (MPO515), since the levels were similar to those in the *cbrXA* mutant (MPO495). This protein retained its transmembrane domains thus it was presumably inserted in the inner membrane as the wild type CbrA. A truncated version of CbrA lacking the 13 TM (ΔTM) domains was not able to yield transcriptional activation of *PP2810* through CbrB when *cbrA* was transcribed from its native promoter (strain MPO508). On the other hand, if the ΔTM-CbrA form was overproduced by directing its expression by a strong P_*tac*_ promoter (strain MPO506), the activity was induced 3.8 fold in OAA compared to succinate, and 5.3-fold compared to LB medium, thus suggesting that this form of CbrA is able to activate transcription in response to limited carbon availability. Nevertheless, its induction levels were 3.4-fold lower in OAA and 3.8-fold lower in succinate than the MPO498 strain complemented with the wild type *cbrXA* sequence (Fig. 8).

**Figure 9:**
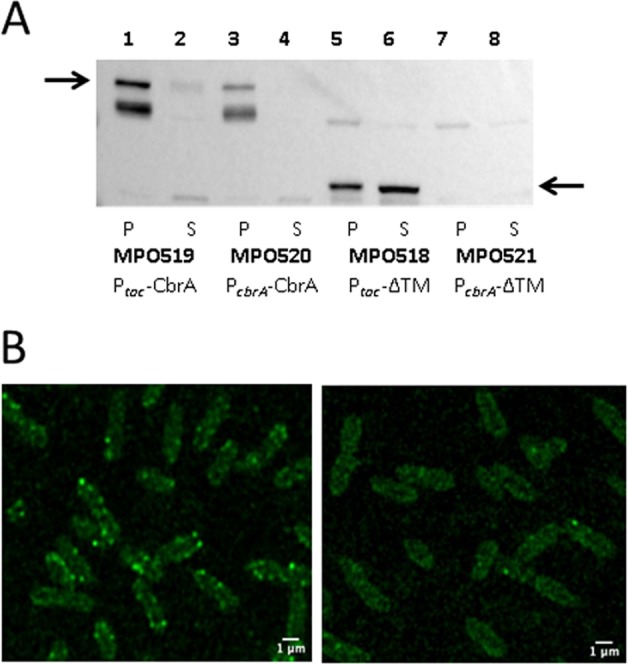
CbrA and ΔTM-CbrA detection by Western blot and confocal microscopy. (**A**) Western blot of soluble and membrane fractions of strains MPO519 (P_*tac*_-CbrA), MPO520 (P_*cbrA*_-CbrA), MPO518 (P_*tac*_-ΔTM) and MPO521 (P_*cbrA*_-ΔTM) grown in a minimal medium with succinate as carbon source. 10 μg of total protein from the unsoluble fractions (lanes 1, 3, 5 and 7) and supernatants (lanes 2, 4, 6 and 8) were loaded onto SDS-PAGE gels. Unsoluble fractions were concentrated 10-fold in order to normalise the amount of total protein loaded in the gel. The black arrows indicate the size corresponding the CbrA- and ΔTM-GFP fusion proteins (136 and 80 kDa, respectively). (**B**) Fluorescence visualisation of strains MPO520 (WT CbrA) (left) and MPO521 (ΔTM-CbrA) (right) with a confocal laser scanning microscope Zeiss LSM 880 equipped with an Airyscan detection unit (63X). Scale bar is indicated in the right. Both pictures obtained from the LCSM were processed with the same parameters

To estimate the amount of wild type and ΔTM-CbrA proteins produced in these strains, and to detect their subcellular localisation, Ct fusions to *gfp* were constructed. The corresponding strains producing GFP fusion proteins were analysed by Western blot for the soluble and membrane protein fractions, and confocal microscopy. Complete CbrA-GFP expressed from its own promoter was clearly produced and associated to the membrane fraction (Fig. 9A, lanes 1 and 3). Overproduction of this protein by using the strong P_*tac*_ promoter resulted in moderately higher production of CbrA-GFP, which was also associated to the membrane fraction. On the other hand, production of the ΔTM-CbrA-GFP version from the P_*cbrA*_ was not readily detectable (Fig. 9A, lanes 7 and 8), while its production was highly increased when expressed from P_*tac*_ (Fig. 9A, lanes 5–6). The ΔTM-CbrA protein overexpressed from P_*tac*_ (Fig. 9A, lane 6) clearly accumulated in the soluble fraction when compared to the full CbrA in the same conditions (Fig. 9A, lane 2). Since the membrane fractions were 10-fold concentrated in relation to the soluble fractions to normalise the amount of total protein loaded onto the Western Blot, a fraction of the ΔTM-CbrA was also visible for the insoluble fraction when overexpressed (Fig. 9A, lane 5). This observation is probably also due to incomplete cell breakage or partial aggregation of the protein in inclusion bodies.

Confocal microscopy of the strain producing CbrA-GFP from its own promoter (MPO520) showed a subcellular localisation of the protein clustered in foci at the surface of the cell, probably inserted in the membrane (Fig. 9B, left). On the other hand, the ΔTM-CbrA produced in the same conditions (MPO521) did not position itself in condensed loci and was dispersed in the cytosol (Fig. 9B, right).

### Overexpression of a ΔTM-CbrA restores the ability to use histidine and citrate as carbon sources

**Figure 10:**
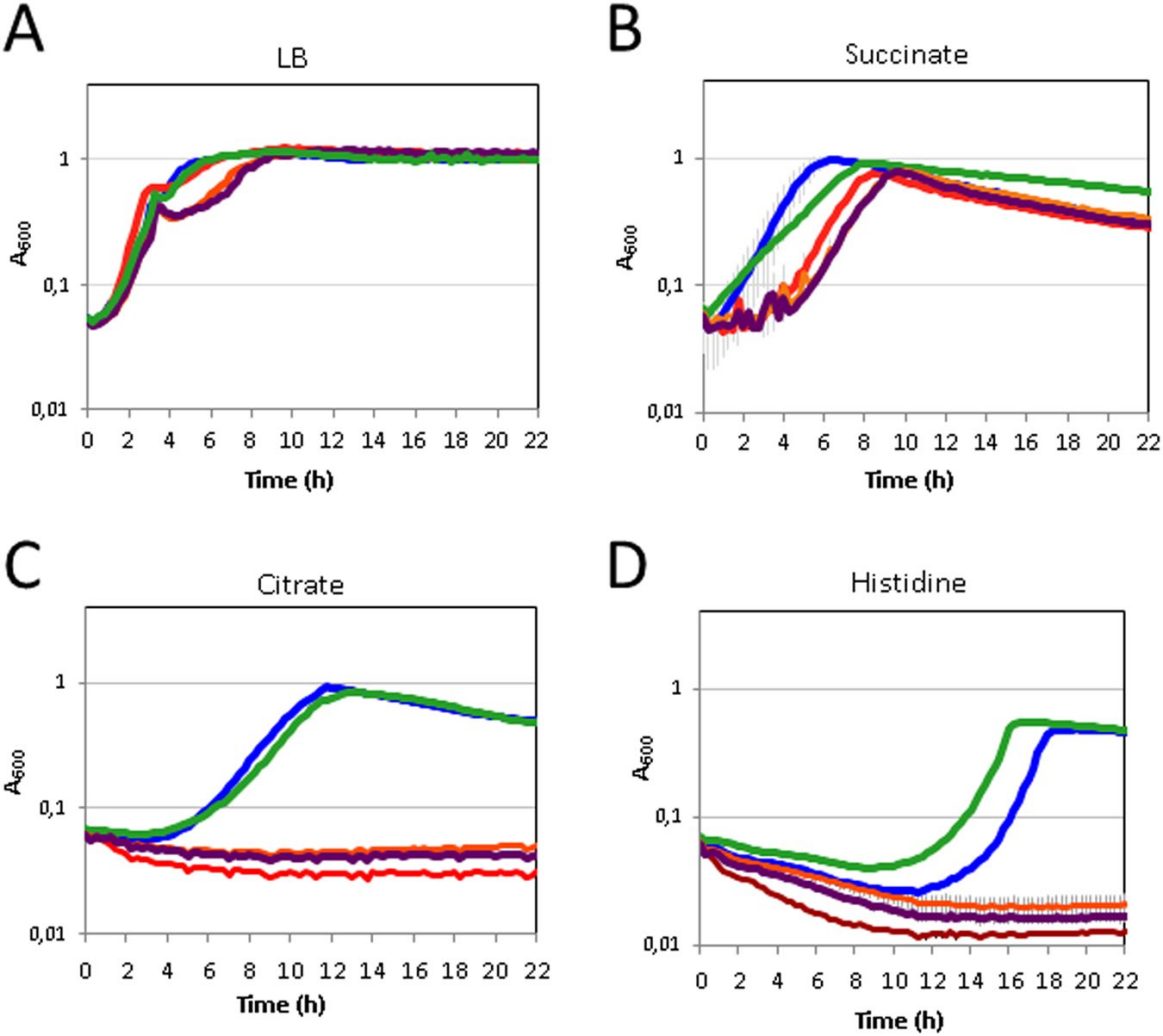
Growth of *P. putida* wild type, Δ*cbrXA*, ΔPAS, ΔTM and P_*tac*_ΔTM mutants in different carbon sources. Growth curves of the wild type strain (MPO451, blue), Δ*cbrXA* mutant (MPO495, red), ΔPAS (MPO507, orange), ΔTM (MPO508, purple) and overexpressed P_tac_ΔTM (MPO506, green) in LB (**A**) and in minimal medium with succinate (**B**), citrate (**C**) and histidine (**D**) as the sole carbon sources. One representative experiment of the three biological replicas is shown, and error bars represent the standard deviation of the three technical replicates for the biological replicate shown.

The putative role of each domain of CbrA in the use of different carbon sources was analysed by monitoring the growth of strains MPO498 (Δ*cbrXA-cbrXA*), MPO495 (Δ*cbrXA*), MPO507 (ΔPAS), MPO508 (ΔTM) and MPO506 (P_tac_ΔTM) for 22 hours of incubation at 30 °C (Fig. 10). None of the variants of CbrA had a considerable defect in their growth on LB or succinate as carbon source, although MPO507 and MPO508 had a longer lag phase reflecting a slightly higher adaptation response. On the other hand, the strains lacking *cbrXA*, or the PAS or TM domains (MPO495, MPO507 and MPO508, respectively) were greatly affected or unable to use citrate or histidine as carbon sources. Overexpression of the C terminal of CbrA containing the PAS-HK domains restored the ability of a *cbrXA* mutant to grow on histidine or citrate as efficiently as the wild type strain, which shows that the TM domains are dispensable for histidine and citrate transport and assimilation (Fig. 10). The relative fluorescence indicative of *PP2810* expression along the timecourse was also monitored in these strains since they also contained the plasmid pMPO355 (*PP2810-gfp* gene fusion). The expression pattern of *PP2810* showed a transient activation during carbon starvation, prior to the start of the growth at the exponential phase for the wild type MPO498 and also for the ΔTM-CbrA MPO506 strains (see Fig. S6). This values were coherent with the previous characterisation of *PP2810* induction measured as the β-galactosidase activity at mid-exponential phase for the overexpressed ΔTM-CbrA strain compared to the ΔTM version in OAA (Fig. 8).

## Discussion

The mechanism of transcriptional regulation by the CbrAB TCS in the *Pseudomonas* and *Azotobacter* groups has been approached as genome wide analysis or as individual target control^11,12,15,19–22,27,28^. However, the activating signal for its induction through the sensor CbrA HK remains unknown. The structural organisation of a putative transmembrane transporter physically linked to the sensor/signalling PAS/autokinase domains, makes CbrA a very interesting component of a new group of HK^17,29^. In this work, we describe the regulatory control of *cbrA* expression and analyse the role its domains in signal detection and CbrA activation of its function.

We have identified a peptide encoded upstream from and overlapping with *cbrA* that directs its translation by a mechanism of translational coupling. This mechanism operates by making translation of a distal gene to depend on the previous translation of the gene immediately upstream^30^ and has been described for instance in processes that have evolved to a maintenance of a correct stoichiometry between the corresponding products. In such cases, independent translation of the distal gene is inhibited, and translation of the proximal gene is able to overcome such inhibition. These include metabolic operons^31–35^, genes encoding ribosomal proteins^36^, chemotaxis^37^, regulatory genes^38^ or a two-component sensor^39^, as *cbrA*. In some systems the translation initiation region (TIR) for the gene downstream is occluded into a secondary structure in the mRNA, that is released upon translation of the distal gene, but in others, the low levels of independent translation of the proximal gene are the consequence of a poor TIR, like in the case of *cbrA*, where there is no recognisable Shine Dalgarno sequence. The fact that a CbrX peptide of the same ATG translation start but different sequence at the non-overlapping sequence to CbrA yields high levels of CbrA (pMPO1259 in Fig. 6) shows that it is not the function of the peptide but its translation *per se* what allows *cbrA* translation.

As the output of a two-component system is the amount of phosphorylated regulator, gene control often use feedback mechanisms to adjust their outputs^1^. A positive or negative retroregulation either controls the amount of regulator, or modify the ability of a sensor or other proteins to alter its phosphorylation. We investigated a possible autoregulatory mechanism for CbrA or an effect on the response regulator CbrB. *cbrB* is transcribed in the same transcriptional unit as *cbrA*, but its expression is also controlled by an internal promoter at the 3′ end of the coding region of *cbrA*^10,20^. We show that the levels of CbrA are regulated in concert with the presence of different carbon sources and are inversely proportional to the carbon availability in the medium, being highest when the carbon source is hardly or not assimilated at all. Nevertheless, CbrA levels are not regulated by neither CbrA or CbrB since they remain unaltered in the *cbrB* and *cbrXA* backgrounds (Fig. 5). In the case of the CbrAB TCS this control is probably exerted by the Carbon catabolite control complex Hfq/Crc^40^. Inspection of the mRNA leader sequence of *cbrXA* revealed that it has a potential Hfq/Crc consensus binding site (AAUAAUAAG)^27,41^ 20 nt downstream of the putative -10 TATA box (Fig. 1). Hfq/Crc prevents the translation of *cbrX*, and indirectly that of *cbrA* through translational coupling. This effect on CbrA is detected in a rich medium (Fig. 5), where there is maximal repression by Hfq/Crc^42^ and the amounts of sRNAs CrcZ and CrcY which titrate Hfq/Crc are lowest^13,21^. In these conditions, Hfq/Crc would repress the translation of *cbrX*, and consequently repression of *cbrA* translation. Some targets, such as *crcZ*, are easily activated by low levels of Cbr activity whilst others, such as PP2810, require more stringent nutrient limiting conditions to be activated (Fig. 4 and ref.^23^). In rich medium many targets would be fully repressed while others may not be sufficiently repressed. Thus, this translational repression of *cbrXA* would constitute an additional regulatory circuit to maintain sufficiently low levels of active CbrA, in order to fully prevent activation of the most sensitive Cbr targets such as *crcZ* in conditions of nutrient abundance. Since *crcZ* in turn prevents the carbon catabolite repression mediated by Crc, its silencing under nutrient abundance might be critical.

CbrA is a member of a unique family of sensor histidine kinases, where its structure suggests that signalling is linked to the transport of a molecule within the same polypeptide^29^. Yet, the mechanism through which CbrA senses and communicates information from the outside is unknown. Zhang *et al*. suggested that CbrA not only senses histidine but is also capable of internalizing it, and that this process is dependent on signalling, and that physical coupling between the TM domain and the C-terminal Histidine kinase of CbrA is required for function^43^. They claim that transport of histidine trough the TM triggers a signal that activate the CbrAB system. Our results on Cbr activation of *PP2810* show that transmembrane domains are not essential for signal perception if supplied at levels sufficiently high (Fig. 8), and that the CbrA activity does not depend on the signal triggered by the transport of histidine or citrate through the TM domains. We also show that the TM domains of CbrA are not essentially required for the uptake of histidine since the strains containing just the ΔTM-CbrA can grow on histidine as the only carbon source, altough they might have the capacity of uptaking histidine into the cell^43^.

Our localisation experiments show that the ΔTM-CbrA accumulates in the cytosol and that the limited activation of the target gene may be at least partially explained by a reduced accumulation of this protein as compared to WT CbrA. However, when overproduction of this soluble protein led to its accumulation to levels sufficiently high, it was possible to detect its activity, and the transcriptional activation of the target gene results dependent on the carbon availability. Therefore, this soluble protein is able to detect the signal, which should be intracellular, and respond to it, probably through its PAS domain. Analogous experiments using HKs chimera of CbrA-CrbS also showed that substrate transport is not needed for signal transduction. The CrbS/R system is a two-component signal transduction system that regulates acetate utilization in *Vibrio cholerae, P. aeruginosa* and *P. entomophila*^29^ and that has a structural disposition similar to CbrA, where a SLC5 domain is linked to the sensor HK domain. However, it differs structurally from CbrA through the REC domain at its C-terminal end of the histidine kinase. The authors show that a chimera containing the PAS-HK domain of CbrA and the 13 TM domains of CrbS recovered the ability to grow on histidine compared to a *cbrAcrbS* deletion mutant, demonstrating in this way the catalytic activity of the PAS-HK domain of CbrA. However, the efficiency of the *Cbr* induction was not quantified^29^. The fusion of a SL50 TM domain to an autokinase domain in signal transduction proteins such as CbrA or CrbS may have a role in early detection of a signal triggering the response that may be outruled when enough catalytic protein is present in the medium. In these cases it seems more plausible that the signal is intracellularly detected from the PAS domain as the interaction with a specific metabolite or a ratio of metabolites as in other HKs sensing C/N ratio, as previously described using a metabolomics approach for CbrB^13^. This hypothesis does not rule out the presence of a potential external signalling through the SL50 TM domains in certain conditions or detection of compounds that are often found at low levels.

## Methods

### Bacterial strains and growth conditions

The bacterial strains used in this work are summarized in Table S1. Cells were grown in M9 minimal medium^44^, containing 20 mM sodium succinate, oxaloacetate, citrate, histidine or glucose as carbon sources, and ammonium chloride (1 g l^−1^) as the nitrogen source. Luria–Bertani (LB) was used as a rich medium^45^. Cultures were grown in culture tubes or flasks with shaking (180 r.p.m.) at 30 °C and 37 °C for *Pseudomonas* or *E. coli* strains, respectively. When required, antibiotics and other additives were used at the following concentrations (μg ml^−1^): ampicillin (Ap) 100; carbenicillin (Cb) 500; kanamycin (Km) 25; rifampicin (Rf) 20; tetracycline (Tc) 5; gentamicin (Gm) 10, 5-bromo-4-chloro-3-indoyl-β-D-galactopyranoside (X-Gal) 25 and isopropyl β-D-thiogalactopyranoside (IPTG) 1 mM. All reagents were purchased from Sigma-Aldrich. Counter-selection for *sacB* carrying strains was done on sucrose agar (10% w/v).

### Construction of the Δ*cbrX-cbrA* deletion strain MPO494

The **Δ***cbrXA* deletion mutant MPO494 was generated by a double event of homologous recombination of plasmid pMPO485 containing the flanking regions of *cbrXA* into *P. putida* KT2442. The procedure was as follows: The left and right flanking regions of *cbrXA* were amplified by PCR from *P. putida* chromosomal DNA with the primer pairs CbrAL1_fwd/CbrAL1_rev and CbrAR_fwd/CbrAR_rev (which generated EcoRI/BamHI sites, and BamHI/HindIII sites, respectively). These 650 and 529 bp DNA fragments were appropriately digested with the corresponding restriction enzymes and cloned into a EcoRI + HindIII-digested pEX18Tc in a 3 fragment ligation, to generate plasmid pMPO484. Then, a 2.5-kbp BamHI-digested fragment from pMPO284 plasmid containing the FRT-flanked kanamycin resistance gene was cloned into the BamHI-digested pMPO484, thus yielding plasmid pMPO485. Plasmid pMPO485 was electroporated into *P. putida* KT2442 and plated onto LB plates containing kanamycin. The co-integrated Km^r^ colonies were tested for tetracyclin sensibility and sucrose resistance and a selected clone was named MPO493, which were verified by PCR using oligonucleotides annealing in the flanking regions of *cbrA*. Plasmid pFLP2 was then conjugated into MPO493 for the excision of the FRT- flanked kanamycin resistance marker^46^ to yield the Δ*cbrX*Δ*cbrA* deletion strain MPO494. Both strains MPO493 and MPO494 were verified by Southern blot analysis (Fig S1).

### Plasmids and strains manipulation

All plasmid DNA preparation and DNA purification kits were purchased from Macherey-Nagel, Promega and General Electric Healthcare and used according to the manufacturers’ specifications. Restriction and modification enzymes were used according to the manufacturers instructions (Roche and NEB). The Klenow fragment or T4 DNA polymerase was routinely used to fill in recessed 3′ ends and trim protruding 3′ ends of incompatible restriction sites. *E*. *coli* DH5α was used as a host in cloning procedures. All cloning steps involving PCR were verified by commercial sequencing (Stab Vida, Portugal). Plasmid DNA was transferred to *E*. *coli* and *P*. *putida* strains by transformation^45^, triparental mating^47^ or electroporation^48^. All plasmids in *E. coli* were transferred by conjugation to *P. putida* in the presence of the helper vector pRK2013. Site-specific integration of miniTn*7* derivatives was performed essentially as described^49^.

### Plasmid construction

Plasmids and oligonucleotides used in this work are summarized in Tables S1 y S2.

Plasmids pMPO355, pMPO356 and pMPO357 were constructed by cloning the promoter regions of *PP2810, crcZ* and *crcY* from pMPO420, pMPO1316 and pMPO1314 as *Eco*RI/*Bam*HI fragments into pMRB1. Plasmids pMPO1370, pMPO1371, pMPO1372, pMPO1373, pMPO1374 and pMPO1259 carrying the wild type sequence of *cbrXA* and point mutations on *cbrX* were constructed by PCR amplification of pMPO1317, pMPO434, pMPO1344, pMPO1349, pMPO1368 and pMPO1369, respectively, using oligonucleotides CbrAcomplF/PcbrAlongSmaI_rev. The corresponding 682-bp fragments were digested with HindIII (blunt ended) and XmaI and subsequently cloned into an EcoRI(blunt ended) + XmaI- digested pMPO200 plasmid. Plasmids pMPO434, pMPO1344 and pMPO1368 for the *cbrX-cbrA* miniTn*7*-based complementation of mutant MPO494 were constructed by overlapping PCR of a 2 kbp DNA fragment of pMPO1317 using the mutagenic oligonucleotides ATGpep_fwd/ATGpep_rev, ATG2pep_fwd/ATG2pep_rev and ATG1-Tpep_fwd/ATG1-Tpep_rev, respectively, and the external non-mutagenic oligonucleotides CbrAcomplF/CbrAEcoRI_rev in a two-step amplification as previously described^50^. The PCR products were digested with and cloned into pMPO1317 digested with the same enzymes to yield pMPO434, pMPO1344 and pMPO1368, respectively. Plasmids pMPO1349 and pMPO1369 were constructed using the mutagenic oligonucleotides ATG2pep_fwd/ATG2pep_rev and Pep3 + C_fwd/Pep3 + C_rev for amplification of plasmids pMPO434 and pMPO1368, respectively, the fragments digested with HindIII + EcoRI and cloned into pMPO1317 digested with the same enzymes. Plasmid pMPO1324 containing a truncated version of *cbrA* lacking the PAS domain (coord. + 1881 to + 2108 from ATG) expressed from its own promoter was generated as follows: a HindIII + SacI digested 2.3-kbp DNA fragment from pMPO1317 containing the *cbrXA* promoter region and a 930-bp fragment generated by PCR amplification of plasmid pMPO1317 with the oligonucleotides CbrA2103fwd/CbrAcomplR digested with SacI/SspI were directionally cloned into HindIII + SmaI-digested pME6182 in a 3 fragment ligation. Plasmid pMPO1325 containing a truncated version of *cbrA* lacking the TM domains (coord. + 4 to + 1575 from ATG) was generated as follows: *a HindIII* + *EcoRI* digested 424-bp DNA fragment obtained from PCR amplification of pMPO1317 with the oligonucleotides CbrAcomplF/CbrA3EcoRev, and an EcoRI + KpnI-digested 1453-bp DNA fragment from plasmid pMPO1317 were directionally cloned into HindIII + KpnI-digested pME6182 in a 3 fragment ligation. Plasmid pMPO358 for integration of a ΔTM-CbrA version expressed under P_*tac*_ in the Tn7 site of the chromosome was constructed as follows: A 1470 bp HindIII + SphI digested PCR fragment from amplification of pMPO483 with oligonucleotides CbrAsol1_fwd/CbrAsol_rev and the 1714 bp fragment bearing the *lacI*^q^-P_*tac*_ expression cassette obtained from pIZ1016 digested with NcoI(blunt) + HindIII, were cloned into the vector pME6182 after restriction with SmaI + SphI in a 3 fragment ligation. Plasmid pMPO1347 containing the ΔPAS-CbrA version with a synthetic Shine Dalgarno sequence obtained with RBS calculator^51^ was constructed by the insertion of a HindIII + NcoI-digested 768 bp PCR amplification fragment from pMPO1324 with oligos CbrATMSD_fwd//CbrATM-NcoI_rev into a pMPO1338 digested with the same enzymes. Plasmid pMPO1338 was generated after partial *Nco*I- digestion of pMPO1324 and religation to inactivate one NcoI site. The P_*tac*_ promoter was inserted into the plasmid pMPO1347 by HindII (blunt) digestion and cloning of the *lacI*^q^-P_*tac*_ expression cassette as a NcoI + HindIII (made blunt) fragment from pIZ1016, to yield plasmid pMPO1348. Plasmid pMPO1350 was generated by PCR amplification of a *gfpmut3* allele in pMRB1 with the oligonucleotidas GFPfusion_fwd/PstI_gfp_rev. The 767-bp resulting DNA fragment digested with SpeI + PstI was cloned into pUC18Sfi-miniTn7BB-Gm, digested with the same enzymes. Subsequently, the 1714-bp DNA fragment containing the *lacI*^*q*^-P_*tac*_ cassette obtained from NcoI + HindIII digestion of plasmid pIZ1016 was cloned into pMPO1350 digested with the same enzymes to generate pMPO1353. Plasmid pMPO1358 containing a ΔTM-CbrA version in high copy number fused to GFP was obtained by the insertion of a PCR generated DNA fragment of 1.48 kbp obtained by amplification of pMPO1325 with oligos CbrAsol2_fwd/CbrAfusionXhoI_rev into the plasmid pMPO1353 after HindIII + XhoI digestion and ligation of both fragments. Plasmid pMPO1261 carrying a *cbrA*’-‘*gfpmut3* in frame fusion expressed from P_*cbrA*_ was constructed by cloning a 1.7-kbp fragment from pMPO1317 digested with HindIII(blunt) + KspI into SpeI(blunt) + KspI-digested pMPO1359. Plasmid pMPO1359 carrying a *cbrA*’-‘*gfpmut3* in frame fusion expressed from P_*tac*_ was constructed by PCR amplification of a 1623-bp, and a 1401-bp PCR fragments with the oligonucleotides CbrATMSD_fwd/CbrAEcoRI_rev and CbrAEcoRI_fwd/CbrAfusionXhoI_rev, respectively, using the plasmid pMPO1317 as template. The PCR products were then cleaved with HindIII + EcoRI and EcoRI + XhoI, respectively, and ligated into HindIII + XhoI-digested pMPO1353. Plasmid pMPO1367 bearing ΔTM*cbrA*’-‘*gfpmut3* fusion was generated by cloning a 1.6-kbp fragment from plasmid pMPO1325 digested with HindIII(blunt) into a SpeI(blunt) + SalI-digested pMPO1359.

### ß-Galactosidase assays

Steady-state β-galactosidase assays were used to examine the expression of *crcZ* and *PP2810* transcriptional fusions and *cbrXA* translational fusions to *lacZ* in *P. putida* KT2442, MPO494 and its derivatives. Preinocula of bacterial strains harbouring the relevant plasmids were grown to saturation in LB or minimal medium with succinate 20 mM as carbon source. Cells were then diluted to 0.05 of A_600_ in LB, to 0.1 of A_600_ in a minimal medium with succinate and to 0.3 of A_600_ in a minimal medium with oxaloacetate and shaken until they reached the mid-exponential phase (A_600_ = 0.25–0.5). β-galactosidase activity was determined from SDS- and chloroform-permeabilised cells as previously described^52^

### GFP quantification for gene expression analysis

Gene expression was also assessed as GFP fluorescence intensity for promoters fused to *gfpmut3-lacZ*. Strains bearing the corresponding fusion plasmids (pMPO355, pMPO356 and pMPO357) were grown overnight in LB and minimal medium, diluted to 0.2 of A_600_ in 150 μl in the same medium and dispensed into the wells of a Costar 96 microtiter polystyrene plate (Corning). The plate was incubated in a TECAN Spark microtiter plate reader/incubator at 30 °C with 510 rpm shaking for 23 hours. For measurement, A_600_ and GFP fluorescence (485 nm excitation, 535 nm emission) were monitored in 15-minute intervals during incubation and the normalised activity was calculated as the ratio between GFP fluorescence intensity and A_600_. For measurements of *crcZ* and *crcY* expression in plasmids pMPO356 and pMPO357, the default gain parameter of 55 was modified to 35.

### RNA purification and RT-PCR

*P. putida* strains were cultivated overnight in LB or minimal medium with succinate as carbon source. The saturated preinocula were diluted to A_600_ = 0.05 in LB, and 0.1 in a minimal medium with succinate and grown to mid-exponencial phase. Since the strains did not grow with OAA as carbon source, the cultures were inoculated at A_600_ = 0.3 and induced for 3 h at 30 °C. The cells were then collected and frozen at −80 °C. RNA extraction and semi-quantitative reverse transcription (RT)-PCR was performed as described previously^21^ with some modifications. cDNAs dilutions (25–0.5 ng) from the experimental samples were amplified using PuRe Taq Ready-To-Go PCR Beads (GE Healthcare) according to the manufacters’ specifications. The detection of transcription segments corresponding to the 5′-end of *cbrA* and *cbrX* were carried out using the pairs of oligonucleotides RT-CbrA_fwd/RT-CbrA_rev and RT-cbrX_fwd/RT-cbrX_rev respectively, resulting in 177-bp amplicons. In adition, cotranscription of *cbrX* and *cbrA* was analysed by RT-PCR using the pairs of oligonucleotides RT-PcbrXA_fwd2/RT-cbrX_rev (oligos 1 and 2 in Fig. 1A), RT-PcbrXA_fwd2//RT-CbrA_rev (oligos 1 and 4 in Fig. 1A) and RT-CbrA_fwd/RT-CbrA_rev (oligos 3 and 4 in Fig. 1A) resulting in the amplification of 207, 414 and 177 bp DNA fragments, respectively. PCR conditions consisted of an initial denaturation step at 94 °C for 5 min, followed by 30 cycles of 94 °C for 30 s, 60 °C for 30 s and 72 °C for 30 s, with a final extesion step 72 °C for 5 min. Negative and positive controls and RT-PCR products obtained were resolved by 2% agarose eletrophoresis gel and visualized by ethidium bromide staining. M corresponds to GeneRuler^TM^ 1 Kb Plus DNA ladder (ThermoFisher Scientific) molecular weight marker.

Quantitative reverse transcription (RT)-PCR of *cbrB* was performed as described previously^53^. RT of 3 μg of total RNA was performed using the High-Capacity cDNA Archive Kit (Applied Biosystems), with random hexamers as primers to generate cDNAs. Target cDNAs (10 ng) from the experimental samples were amplified in triplicate in separate PCR reactions using 0.3 mM of each primer cbrB23 1Q/cbrB 74 2Q as previously described^21^.

### Western blot

The strains of *P. putida* KT2442, MPO520, MPO521, MPO519 and MPO518 were induced in minimal medium with succinate as carbon source as described before. Cell cultures were washed three times with cold phosphate sodium buffer supplemented with protease inhibitors, collected by centrifugation at 4000 g for 20 min at 4 °C and resuspended in 1 ml of the same buffer. The cells were disrupted by sonication by 12 pulses of 2–3 seconds each, and the cell lysate was centrifuged at 13000 g for 2 min. The supernatant was collected and the pellet was washed twice, and concentrated 10-fold into 100 μl of 8 M Urea, 0.1% SDS buffer for sonication as described above. Protein concentrations from the soluble and unsoluble fractions were determined using the RC-DC Protein Assay (Biorad). 10 μg of total protein from each fraction were loaded for SDS-PAGE, and transferred to nitrocellulose membranes with a Biorad Trans-Blot system (TGX Stain-Free “Fast-Cast” Acrylamide kit). The membranes were washed with TBST 1 × (Tris Buffer Saline 10 × , 0,1% Tween-20) and blocked for 1 h at room temperature with 5% (w/v) skimmed milk powder in TBST 1 × . Antibodies anti-GFP (1:2000) (Invitrogen) were used for detection and were incubated with the blots overnight at 4 °C with gentle shaking. Peroxidase-labeled secondary antibodies (1:10000) were added, and the membranes were incubated at 4 °C for 1 h then washed with TBST 1X four times for 15 min. Transference of immunoreactive products to anti-GFP were detected by the enhanced chemiluminescense system (SuperSignal West Dura Extended Duration Substrate, Thermo Scientific). *P. putida* KT2442 extracts were used as a control to confirm the absence of fusion protein.

### Confocal microscopy

Cell cultures grown in minimal medium with succinate as carbon source were incubated for 3 h to mid-exponential phase (A_600_ = 0,3). 0.5 ml each culture were incubated on 35-mm glass bottom culture dishes (MatTek) for 15 min, washed twice gently with phosphate sodium buffer 1X and fixed with ρ-formaldehyde (PFA) 4% during 10 min. Preparations were visualised by confocal laser scanning ZEISS LSM 880 (Carl Zeiss AG, Oberkochen, Germany) equipped with an Airyscan detection unit. To maximize the resolution enhancement a 63 × /1.46 NA oil immersion lens were used. An argon laser, at 488 nm, was used as the excitation source for the fluorescent probe. Maximum intensity projections of 5 z–stacks were analysed using ImageJ (Schneider, Rasband, & Eliceiri, 2012).Image preprocessing comprised linear adjustments as brightness and constrast followed by applying a Gaussian blur filter radius 1.5 and subsequently by a sharpening algorithm.

## Supplementary information

Supplementary Figures and Tables

## References

[1] Groisman EA (2016). Feedback Control of Two-Component Regulatory Systems. Annu Rev Microbiol.

[2] Martín-Mora David, Fernández Matilde, Velando Félix, Ortega Álvaro, Gavira José, Matilla Miguel, Krell Tino (2018). Functional Annotation of Bacterial Signal Transduction Systems: Progress and Challenges. International Journal of Molecular Sciences.

[3] Krell T (2010). Bacterial sensor kinases: diversity in the recognition of environmental signals. Annu Rev Microbiol.

[4] Mascher T (2014). Bacterial (intramembrane-sensing) histidine kinases: signal transfer rather than stimulus perception. Trends Microbiol.

[5] Krell T (2015). Tackling the bottleneck in bacterial signal transduction research: high-throughput identification of signal molecules. Mol Microbiol.

[6] Vreede J, van der Horst MA, Hellingwerf KJ, Crielaard W, van Aalten DM (2003). PAS domains. Common structure and common flexibility. J Biol Chem.

[7] Aguilar PS, Hernandez-Arriaga AM, Cybulski LE, Erazo AC, de Mendoza D (2001). Molecular basis of thermosensing: a two-component signal transduction thermometer in Bacillus subtilis. EMBO J.

[8] Mascher T, Margulis NG, Wang T, Ye RW, Helmann JD (2003). Cell wall stress responses in Bacillus subtilis: the regulatory network of the bacitracin stimulon. Mol Microbiol.

[9] Li W, Lu CD (2007). Regulation of carbon and nitrogen utilization by CbrAB and NtrBC two-component systems in Pseudomonas aeruginosa. J Bacteriol.

[10] Nishijyo, T., Haas, D. & Itoh, Y. The CbrA-CbrB two-component regulatory system controls the utilization of multiple carbon and nitrogen sources in Pseudomonas aeruginosa. *Mol Microbiol* **40**, 917–931, doi:mmi2435 [pii] (2001). 10.1046/j.1365-2958.2001.02435.x 11401699

[11] Quiroz-Rocha E (2017). Two-component system CbrA/CbrB controls alginate production in Azotobacter vinelandii. Microbiology.

[12] Quiroz-Rocha E (2017). Glucose uptake in Azotobacter vinelandii occurs through a GluP transporter that is under the control of the CbrA/CbrB and Hfq-Crc systems. Sci Rep.

[13] Valentini M (2014). Hierarchical management of carbon sources is regulated similarly by the CbrA/B systems in Pseudomonas aeruginosa and Pseudomonas putida. Microbiology.

[14] Yeung, A. T., Bains, M. & Hancock, R. E. The sensor kinase CbrA is a global regulator that modulates metabolism, virulence, and antibiotic resistance in Pseudomonas aeruginosa. *J Bacteriol* **193**, 918–931, doi:JB.00911-10 [pii]10.1128/JB.00911-10 (2011). 10.1128/JB.00911-10 PMC3028677 21169488

[15] Amador CI, Canosa I, Govantes F, Santero E (2010). Lack of CbrB in Pseudomonas putida affects not only amino acids metabolism but also different stress responses and biofilm development. Environ Microbiol.

[16] Jung Kirsten, Fabiani Florian, Hoyer Elisabeth, Lassak Jürgen (2018). Bacterial transmembrane signalling systems and their engineering for biosensing. Open Biology.

[17] Korycinski M (2015). STAC–A New Domain Associated with Transmembrane Solute Transport and Two-Component Signal Transduction Systems. J Mol Biol.

[18] Muzhingi, I. *et al*. Modulation of CrbS-dependent activation of the acetate switch in Vibrio cholerae. *J Bacteriol*, 10.1128/JB.00380-18 (2018). 10.1128/JB.00380-18 PMC6222196 30224439

[19] Abdou, L., Chou, H. T., Haas, D. & Lu, C. D. Promoter recognition and activation by the global response regulator CbrB in Pseudomonas aeruginosa. J Bacteriol **193**, 2784–2792, doi:JB.00164-11 [pii]10.1128/JB.00164-11 (2011). 10.1128/JB.00164-11 PMC3133114 21478360

[20] Amador Cristina I., López- Sánchez Aroa, Govantes Fernando, Santero Eduardo, Canosa Inés (2016). APseudomonas putida cbrBtransposon insertion mutant displays a biofilm hyperproducing phenotype that is resistant to dispersal. Environmental Microbiology Reports.

[21] Garcia-Maurino SM, Perez-Martinez I, Amador CI, Canosa I, Santero E (2013). Transcriptional activation of the CrcZ and CrcY regulatory RNAs by the CbrB response regulator in Pseudomonas putida. Mol Microbiol.

[22] Sonnleitner E (2012). Novel targets of the CbrAB/Crc carbon catabolite control system revealed by transcript abundance in Pseudomonas aeruginosa. PLoS One.

[23] Barroso R (2018). The CbrB Regulon: Promoter dissection reveals novel insights into the CbrAB expression network in Pseudomonas putida. PLoS One.

[24] Linares, J. F. *et al*. The global regulator Crc modulates metabolism, susceptibility to antibiotics and virulence in Pseudomonas aeruginosa. *Environ Microbiol* **12**, 3196–3212, doi:EMI2292 [pii]10.1111/j.1462-2920.2010.02292.x (2010). 10.1111/j.1462-2920.2010.02292.x 20626455

[25] Moreno, R., Fonseca, P. & Rojo, F. The Crc global regulator inhibits the Pseudomonas putida pWW0 toluene/xylene assimilation pathway by repressing the translation of regulatory and structural genes. *J Biol Chem* **285**, 24412–24419, doi:M110.126615 [pii]10.1074/jbc.M110.126615 (2010). 10.1074/jbc.M110.126615 PMC2915677 20529863

[26] Moreno R (2015). The Crc and Hfq proteins of Pseudomonas putida cooperate in catabolite repression and formation of ribonucleic acid complexes with specific target motifs. Environ Microbiol.

[27] Sonnleitner E, Abdou L, Haas D (2009). Small RNA as global regulator of carbon catabolite repression in Pseudomonas aeruginosa. Proc Natl Acad Sci USA.

[28] La Rosa R, Nogales J, Rojo F (2015). The Crc/CrcZ-CrcY global regulatory system helps the integration of gluconeogenic and glycolytic metabolism in Pseudomonas putida. Environ Microbiol.

[29] Sepulveda E, Lupas AN (2017). Characterization of the CrbS/R Two-Component System in Pseudomonas fluorescens Reveals a New Set of Genes under Its Control and a DNA Motif Required for CrbR-Mediated Transcriptional Activation. Front Microbiol.

[30] McCarthy JE, Gualerzi C (1990). Translational control of prokaryotic gene expression. Trends Genet.

[31] Oppenheim DS, Yanofsky C (1980). Translational coupling during expression of the tryptophan operon of Escherichia coli. Genetics.

[32] Schumperli D, McKenney K, Sobieski DA, Rosenberg M (1982). Translational coupling at an intercistronic boundary of the Escherichia coli galactose operon. Cell.

[33] Rex G, Surin B, Besse G, Schneppe B, McCarthy JE (1994). The mechanism of translational coupling in Escherichia coli. Higher order structure in the atpHA mRNA acts as a conformational switch regulating the access of de novo initiating ribosomes. J Biol Chem.

[34] Govantes F, Andujar E, Santero E (1998). Mechanism of translational coupling in the nifLA operon of Klebsiella pneumoniae. EMBO J.

[35] Little S, Hyde S, Campbell CJ, Lilley RJ, Robinson MK (1989). Translational coupling in the threonine operon of Escherichia coli K-12. J Bacteriol.

[36] Baughman G, Nomura M (1983). Localization of the target site for translational regulation of the L11 operon and direct evidence for translational coupling in Escherichia coli. Cell.

[37] Lovdok L (2009). Role of translational coupling in robustness of bacterial chemotaxis pathway. PLoS Biol.

[38] Dubytska, L. Borrelia burgdorferi 297 bmpA encode the mRNA that contains ORF for a leader peptide that regulates bmpA gene expression. *bioRxiv*, doi:http://dx.doi.org/10.1101/542589. (2019).

[39] Liljestrom P, Laamanen I, Palva ET (1988). Structure and expression of the ompB operon, the regulatory locus for the outer membrane porin regulon in Salmonella typhimurium LT-2. J Mol Biol.

[40] Hernandez-Arranz S, Sanchez-Hevia D, Rojo F, Moreno R (2016). Effect of Crc and Hfq proteins on the transcription, processing, and stability of the Pseudomonas putida CrcZ sRNA. RNA.

[41] Moreno, R., Marzi, S., Romby, P. & Rojo, F. The Crc global regulator binds to an unpaired A-rich motif at the Pseudomonas putida alkS mRNA coding sequence and inhibits translation initiation. *Nucleic Acids Res* **37**, 7678–7690, doi:gkp825 [pii]10.1093/nar/gkp825 (2009). 10.1093/nar/gkp825 PMC2794181 19825982

[42] Moreno R, Fonseca P, Rojo F (2012). Two small RNAs, CrcY and CrcZ, act in concert to sequester the Crc global regulator in Pseudomonas putida, modulating catabolite repression. Mol Microbiol.

[43] Zhang XX, Gauntlett JC, Oldenburg DG, Cook GM, Rainey PB (2015). Role of the Transporter-Like Sensor Kinase CbrA in Histidine Uptake and Signal Transduction. J Bacteriol.

[44] Mandelbaum RT, Wackett LP, Allan DL (1993). Mineralization of the s-triazine ring of atrazine by stable bacterial mixed cultures. Appl Environ Microbiol.

[45] Sambrook, J., E. F. Fritsch, and T. Maniatis *Molecular cloning: a laboratory manual*. (Cold Spring Harbor Laboratory Press, 2000).

[46] Hoang TT, Karkhoff-Schweizer RR, Kutchma AJ, Schweizer HP (1998). A broad-host-range Flp-FRT recombination system for site-specific excision of chromosomally-located DNA sequences: application for isolation of unmarked Pseudomonas aeruginosa mutants. Gene.

[47] Figurski DH, Helinski DR (1979). Replication of an origin-containing derivative of plasmid RK2 dependent on a plasmid function provided in trans. Proc Natl Acad Sci USA.

[48] Choi KH, Kumar A, Schweizer HP (2006). A 10-min method for preparation of highly electrocompetent Pseudomonas aeruginosa cells: application for DNA fragment transfer between chromosomes and plasmid transformation. J Microbiol Methods.

[49] Choi KH (2005). A Tn7-based broad-range bacterial cloning and expression system. Nat Methods.

[50] Camacho EM, Casadesus J (2005). Regulation of traJ transcription in the Salmonella virulence plasmid by strand-specific DNA adenine hemimethylation. Mol Microbiol.

[51] Salis HM (2011). The ribosome binding site calculator. Methods Enzymol.

[52] Miller, J. H. *A short course in bacterial genetics: a laboratory manual*. (Cold Spring Harbor Laboratory Press, 1992).

[53] Yuste, L. *et al*. Growth phase-dependent expression of the Pseudomonas putida KT2440 transcriptional machinery analysed with a genome-wide DNA microarray. *Environ Microbiol* **8**, 165–177, doi:EMI890 [pii]10.1111/j.1462-2920.2005.00890.x (2006). 10.1111/j.1462-2920.2005.00890.x 16343331

