# Supplementary material for: Unraveling the role of the CbrA histidine kinase in the signal transduction of the CbrAB two-component system in Pseudomonas putida
Source: Sci Rep. 2019 Jun 24;9:9110. doi: 10.1038/s41598-019-45554-9 (PMC6591292; doi:10.1038/s41598-019-45554-9)
Supplement: Supplementary file 1 — Supplementary Figures and Tables [file 41598_2019_45554_MOESM1_ESM.pdf]

Supplementary Material (Figures S1- S6, Tables S1-S2)

Unraveling the role of the CbrA histidine kinase in the signal transduction of the CbrAB two component system in *Pseudomonas putida*

Elizabet Monteagudo-Cascales[1], Sofía M. García-Mauriño[1], Eduardo Santero[1] and Inés Canosa[1]*

[1] Universidad Pablo de Olavide, Centro Andaluz de Biología del Desarrollo/ Consejo Superior de Investigaciones Científicas/ Junta de Andalucía, Seville, Spain. E-mail: icanper@upo.es

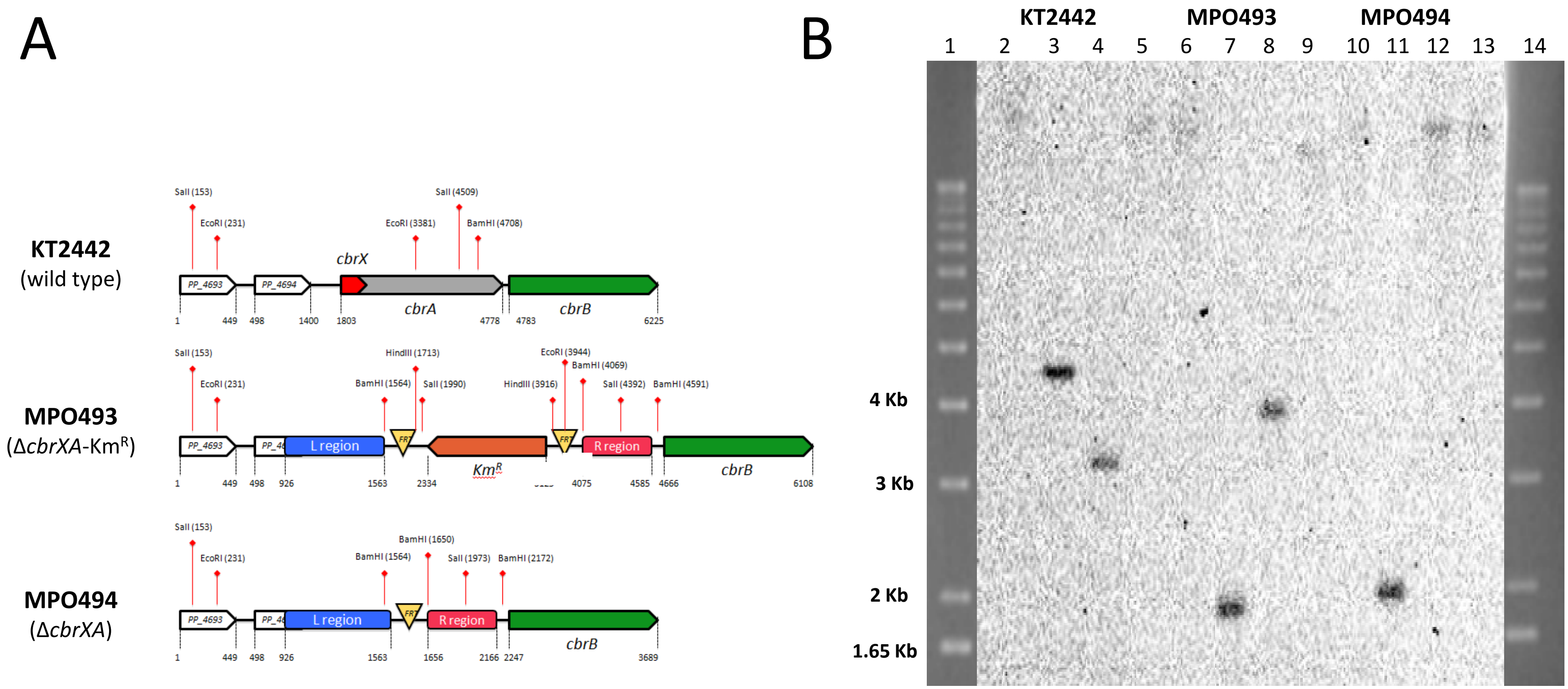

Figure S1. Southern blot of a Δ*cbrX-cbrA* mutant (MPO494) strain. **(A)** Diagram showing the genomic context for the wild type *cbrX-cbrA-cbrB* locus, the deletion mutant containing the FRT-Km-FRT cassete (MPO493) and Δ*cbrX-cbrA* deletion mutant with the excised FRT-Km-FRT cassete (MPO494). Restriction sites and coordinates are represented as red vertical markers and in parenthesis, respectively. The upstream DNA sequence of the deleted gene is depicted in blue as L-region, and the downstream region of the gene in pink as R-region. The FRT-sites are represented as inverted yellow triangles. The diagram is not shown to scale. **(B)** Southern blot analysis of *P. putida* KT2442, MPO493 and MPO494. Lanes 1 and 14 are Molecular weight ladders. Digestion of genomic DNA of the strains are migrated in agarose for BamHI- (lanes 2, 6 and 10), SalI- (lanes 3, 7 and 11), EcoRI- (lanes 4, 8 and 12) and HindIII- (lanes 5, 9 and 13). The transferred membrane was hybridised with a DIG-labeled DNA fragment containing a L-region annealing upstream of *cbrX-cbrA*. The expected fragment size for SalI digestion were 4.3, 1.8 and 1.8-kbp to KT2442, MPO493 and MPO494, respectively, and for EcoRI digestion were 3.1 and 3.7-kbp for KT2442 and MPO493, respectively.

A

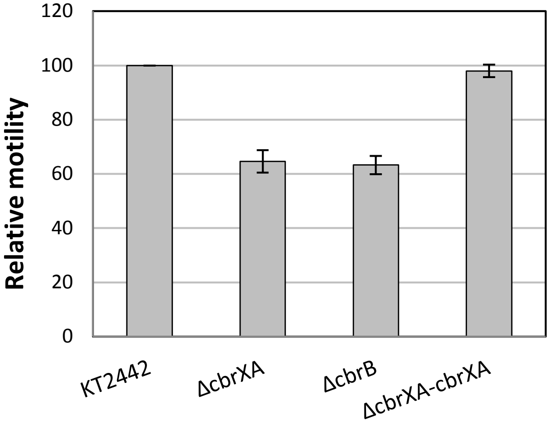

B

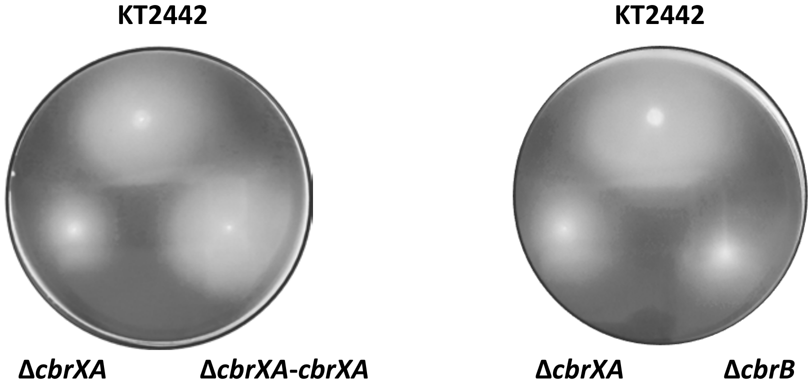

Figure S2. Swimming motility assays. (A) Quantification of the relative swimming motility in semisolid LB agar for strains MPO494 (Δ*cbrXA*) and MPO401 (Δ*cbrB*) and MPO498 (Δ*cbrXA-cbrXA*) compared with the wild type KT2442. The data are mean values of at least three biological replicates, with their corresponding standard deviation. Plates were incubated at 30ºC for 24 h before motility was scored. (**B)** A representative replicate of the motility assay is shown.

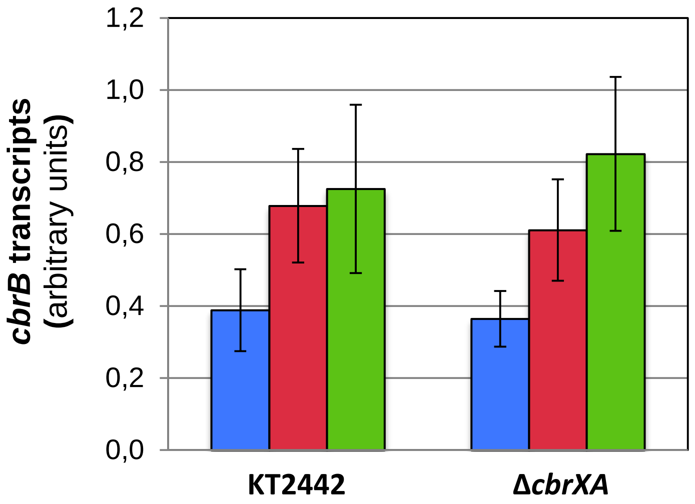

Figure S3. Levels of *cbrB* mRNA - RT-qPCR quantification of cbrB transcripts in a KT2442 wild type and MPO494 (ΔcbrXA) mutant starain in LB (blue) or a minimal medium containind succinate (red) or OAA (green) as carbon sources.

pMPO1370 (*wt*)

ATGTACATCTATCGTTTGGTCCTGCTTCTGGTCGTGGGGATCTACCTGTTCTCCCCGGCCATC**ATG**GACTGGTGGATCGAACCGACCGGA
GCCTGGTACCGCCCCTACCTGCTCTGGCTGATCCTGATCGTCGTCACCTTCATCCTGCAGAGCCAACGAG**ATG**CCGATGAGCTT**TAG**…

MYIYRLVLLLVVGIYLFSPAIMDWWIEPTGAWYRPYLLWLILIVVTFILQSQRDADEL-

pMPO1371 (*atg1*)

GCATACATCTATCGTTTGGTCCTGCTTCTGGTCGTGGGGATCTACCTGTTCTCCCCGGCCATC**ATG**GACTGGTGGATCGAACCGACCGGA
GCCTGGTACCGCCCCTACCTGCTCTGGCTGATCCTGATCGTCGTCACCTTCATCCTGCAGAGCCAACGAG**ATG**CCGATGAGCTT**TAG**…

*A*YIYRLVLLLVVGIYLFSPAIMDWWIEPTGAWYRPYLLWLILIVVTFILQSQRDADEL-

pMPO1372 (*atg2*)

ATGTACATCTATCGTTTGGTCCTGCTTCTGGTCGTGGGGATCTACCTGTTCTCCCCGGCCATC**GAT**GACTGGTGGATCGAACCGACCGGA
GCCTGGTACCGCCCCTACCTGCTCTGGCTGATCCTGATCGTCGTCACCTTCATCCTGCAGAGCCAACGAG**ATG**CCGATGAGCTT**TAG**…

AYIYRLVLLLVVGIYLFSPAIMDWWIEPTGAWYRPYLLWLILIVVTFILQSQRDADEL-

pMPO1373 (*atg1&2*)

GCATACATCTATCGTTTGGTCCTGCTTCTGGTCGTGGGGATCTACCTGTTCTCCCCGGCCATC**GAT**GACTGGTGGATCGAACCGACCGGA
GCCTGGTACCGCCCCTACCTGCTCTGGCTGATCCTGATCGTCGTCACCTTCATCCTGCAGAGCCAACGAG**ATG**CCGATGAGCTT**TAG**…

*A*YIYRLVLLLVVGIYLFSPAI*D*DWWIEPTGAWYRPYLLWLILIVVTFILQSQRDADEL-

pMPO1374 (*-1*)

ATG _ACATCTATCGTTTGGTCCTGCTTCTGGTCGTGGGGATCTACCTGTTCTCCCCGGCCATC**ATG**GACTGGTGGATCGAACCGACCGGA
GCCTGGTACCGCCCCTACCTGCTCTGGCTGATCCTGATCGTCGTCACCTTCATCCTGCAGAGCCAACGAG**ATG**CCGATGAGCTT**TAG**…

M*TSIVWSCFWSWGSTCSPRPSWTGGSNRPEPGTAPTCSG*-

pMPO1259 (*-1+1*)

ATG _ACATCTATCGTTTGGTCCTGCTTCTGGTCGTGGGGATCTACCTGTTCTCCCCGGCCATC**ATG**GACTGGTGGATCGAACCGACCGGA
GCCTGGTACCGCCCCTACCTGCTCTGGC**C**TGATCCTGATCGTCGTCACCTTCATCCTGCAGAGCCAACGAG**ATG**CCGATGAGCTT**TAG**…

M*TSIVWSCFWSWGSTCSPRPSWTGGSNRPEPGTAPTCSG***LILIVVTFILQSQRDADEL**-

Figure S4. DNA sequence of the point mutagenesis of *cbrX* in plasmids. DNA sequences for *cbrX* and 5’ of *cbrA* in plasmids pMPO1370 (wild type) pMPO1317 (atg1) containing mutated ATG1, pMPO1372 (atg2) containing a mutation in the second in-phase ATG, pMPO1373 (atg1&2) with mutations at ATG1 and ATG2, pMPO1374 (-1) bearing a T deletion at position +4 from ATG1, and pMPO1259 (-1+1), where pMPO1374 recovers the original reading frame by a C insertion at position +118. Substitutions are denoted in red, deletions are represented as a red dash (_), the start and stop codons for *cbrX* and *cbrA* are in bold and underlined. The *cbrX* sequence overlapping with *cbrA* is indicated in dark blue. The predicted amino acid sequence of translated peptides are indicated in pink below each DNA sequence. Residues corresponding to CbrX in its wild type reading frame are shown in bold while amino acids in italic are residues not coincident with the wild type CbrX sequence.

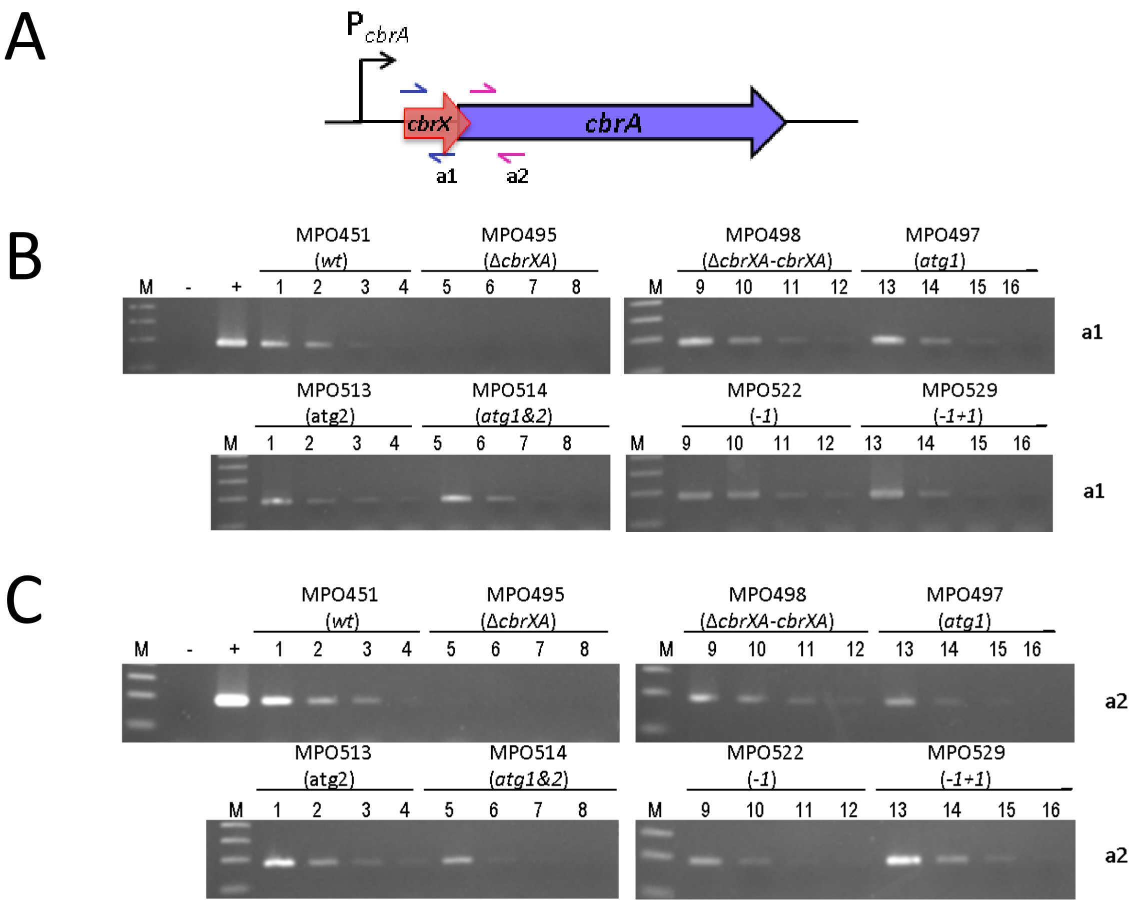

Figure S5. RT-PCR analysis of the *cbrXcbrA* transcriptional unit. (A) Diagram showing the genomic context for *cbrX-cbrA* and the amplicons generated by RT-PCR for oligonucleotides annealing at *cbrX* and *cbrA*. 176 bp DNA fragment generated by RT-PCR using oligonucleotides RT-cbrX_fwd/RT-cbrX_rev annealing at the *cbrX* coding region (coord. -160 to +17 from ATG *cbrA*) (a1, blue arrows) **(B),** and primers RT-CbrA_fwd/RT-CbrA_rev within *cbrA* (coord. +48 to +224 from ATG *cbrA*) (a2, pink arrows) **(C).** cDNA was obtained from MPO451 (wild type), MPO495 (Δ*cbrXA*), MPO498 (Δ*cbrXA*-*cbrXA*), MPO497 (Δ*cbrXA*-ATG1), MPO513 (Δ*cbrXA*-ATG2), MPO514 (Δ*cbrXA*-ATG1&2), MPO522 (Δ*cbrXA*-ΔT) and MPO529 (Δ*cbrXA*-ΔT+C) cultures grown on minimal media with oxaloacetate as carbon source. The amount of cDNA in nanograms used as template for each strain were 25 ng (lanes 1, 5, 9, 13), 5 ng (lanes 2, 6, 10, 14), 1 ng (lanes 3, 7, 11, 15) and 0.5 ng (lanes 4, 8, 12, 16). No DNA as negative control (-) and 50 ng *P. putida* KT2442 genomic DNA was used as positive control (+).

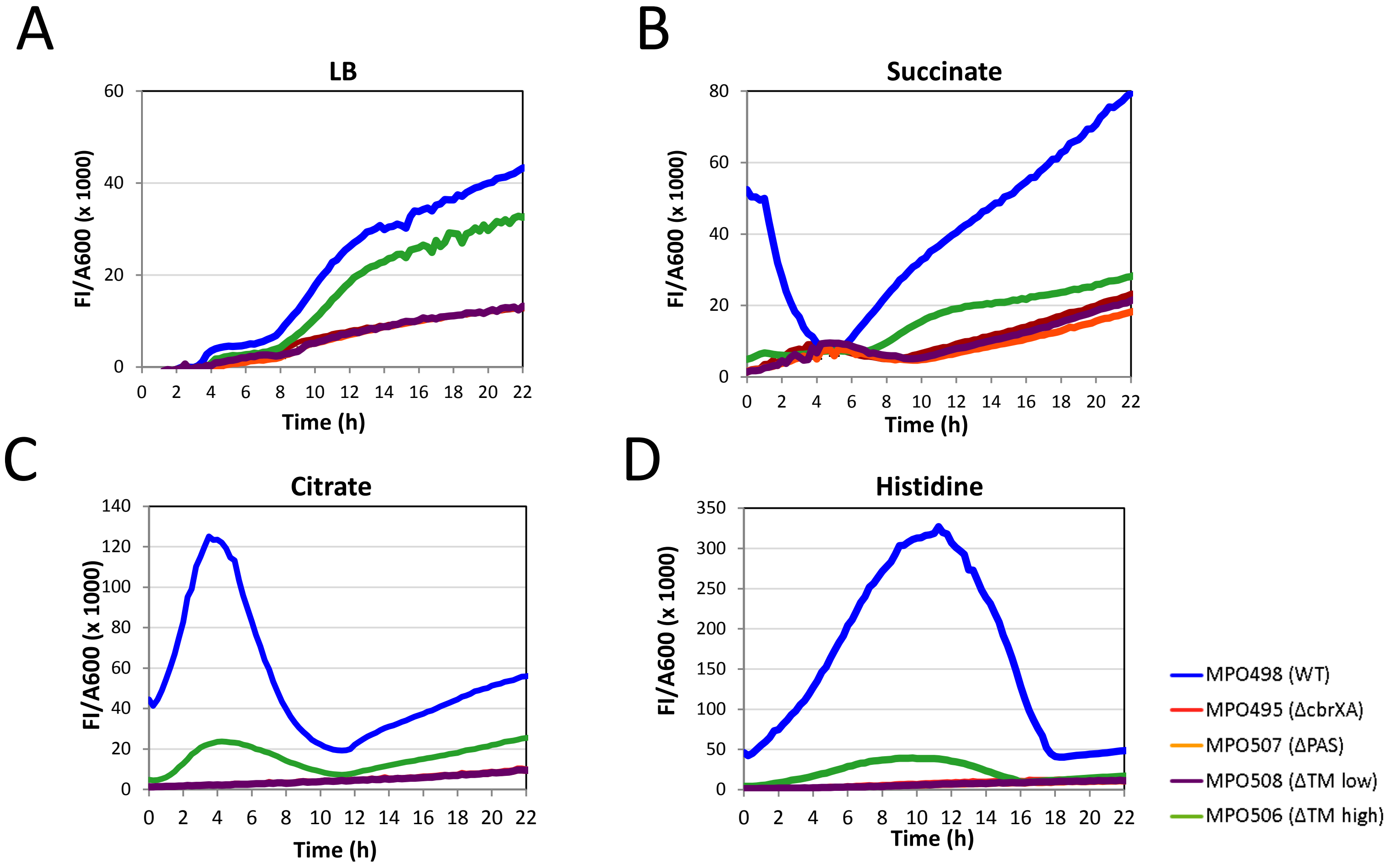

Figure S6. Expression of *P. putida* wild type, *cbrXA*, PAS, TM and P_{tac}TM mutants in different carbon sources. The expression levels are represented as the fluorescence intensity (FI) detected from *gfp* normalised by the optical density at A_{600} for strains MPO498 (Δ*cbrXA*-*cbrXA*, blue), MPO495 (Δ*cbrXA*, red), ΔPAS (MPO507, orange), ΔTM (MPO508, purple) or P_{tac}ΔTM (MPO506, green light), bearing a transcriptional fusion of *PP2810* to *gfpmut3-lacZ* in plasmid pMPO355. The expression levels are monitored for 22 hours in cultures grown in a LB rich medium **(A)** and in a minimal medium containing succinate **(B),** citrate **(C)** and histidine **(D).** One representative experiment with three biological replicates is shown. Error bars represent the standard deviation of the three technical replicates.

Supplementary Tables

Table S1. Strains and plasmids used in this study

Strains	Genotype	Plasmid used for strain generation	Reference/ source
E. coli			
DH5α	*recA1 endA1 hsdR17 supE44 thi-1 gyrA96 relA1*D (*lacZYA-argF* U169 [ϕ80d*lacZDM15*] F$^-$ Nal^r)		[1]
P. putida			
KT2442	mt-2 *hsdR*1(r$^-$ m$^+$) Rif^r		[2]
KT2442-C1	KT2442 *crc*::tet, Tc^r Rif^r		[3]
MPO451	KT2442 miniTn7BB-Gm::*glmS*, Gm^r, Rif^r		[4]
MPO493	KT2442, Δ*cbrXA*::Km, Km^r, Rif^r	[pMPO485]	This work
MPO494	MPO493 FRT- Km excised, Rif^r		This work
MPO495	MPO494 miniTn7BB-Gm::*glmS*, Gm^r, Rif^r	[pME6182]	This work
MPO497	MPO494 miniTn7BB-Gm[P_{cbrA}-*cbrX* (ATG1)-*cbrA*]::*glmS*, Gm^r, Rif^r	[pMPO434]	This work
MPO498	MPO494 miniTn7BB-Gm[P_{cbrA}-*cbrX*-*cbrA*]::*glmS*, Gm^r, Rif^r	[pMPO1317]	This work
MPO506	MPO494 miniTn7BB-Gm[P_{tac}-*cbrA* ΔTM]::*glmS*, Gm^r, Rif^r	[pMPO358]	This work
MPO507	MPO494 miniTn7BB-Gm[P_{cbrA}-*cbrA* ΔPAS]::*glmS*, Gm^r, Rif^r	[pMPO1324]	This work
MPO508	MPO494 miniTn7BB-Gm[P_{cbrA}-*cbrA* ΔTM]:: *glmS*, Gm^r, Rif^r	[pMPO1325]	This work
MPO513	MPO494 miniTn7BB-Gm[P_{cbrA}-*cbrX* (ATG2)-*cbrA*]::*glmS*, Gm^r, Rif^r	[pMPO1344]	This work
MPO514	MPO494 miniTn7BB-Gm[P_{cbrA}-*cbrX* (ATG1&2)-*cbrA*]::*glmS*, Gm^r, Rif^r	[pMPO1349]	This work
MPO515	MPO494 miniTn7BB-Gm[P_{tac}-*cbrA* ΔPAS]::*glmS*, Gm^r, Rif^r	[pMPO1348]	This work
MPO518	MPO494 miniTn7BB-Gm[P_{tac}-*cbrA* ΔTM::*gfpmut3*]::*glmS*, Gm^r, Rif^r	[pMPO1358]	This work
MPO519	MPO494 miniTn7BB-Gm[P_{tac}-*cbrA*::*gfpmut3*]::*glmS*, Gm^r, Rif^r	[pMPO1359]	This work
MPO520	MPO494 miniTn7BB-Gm[P_{cbrA}-*cbrA*::*gfpmut3*]::*glmS*, Gm^r, Rif^r	[pMPO1261]	This work
MPO521	MPO494 miniTn7BB-Gm[P_{cbrA}-*cbrA* ΔTM::*gfpmut3*]::*glmS*, Gm^r, Rif^r	[pMPO1367]	This work
MPO522	MPO494 miniTn7BB-Gm[P_{cbrA}-*cbrX* (ΔT)-*cbrA*]::*glmS*, Gm^r, Rif^r	[pMPO1368]	This work
MPO529	MPO494 miniTn7BB-Gm[P_{cbrA}-*cbrX* (ΔT+C)-*cbrA*]::*glmS*, Gm^r, Rif^r	[pMPO1369]	This work

Plasmids	Phenotype	Reference/source
pEX18Tc	Conjugative plasmid for gene replacement with MCS from pUC18. Tc^r, Mob^+, oriT, *sacB*$^+$	[5]
pFLP2	FLP recombinase expression plasmid. Ap^r	[5]
pIZ1016	Broad-host range expression vector bearing *lacI*^{q} and P_{tac}. Gm^r	[6]
pME6182	Mini-Tn*7* gene delivery vector based on pME3280a, *Hind*III-*Sma*I-*Kpn*I-*Nco*I-*Sph*I cloning site, ColE1 replicon. Ap^r Gm^r	[7]
pMPO200	Broad-host-range *lacZ* protein fusion vector based on pBBR1MCS-4. Ap^r	[8]
pMPO234	Broad-host-range *lacZ* transcriptional fusion vector, based on pMPO200. Ap^r	[9]
pMPO284	pPS854-derived vector containing the pUTminiTn*5*-Km Km^r gene flanked by FRT sites. Ap^r Km^r	[10]
pMPO355	pMRB1-derived vector with the transcriptional fusion of P_{PP2810}::*gfpmut3-lacZ* (coord. -472 and -10 from TSS) cloned as *Eco*RI/*Bam*HI. Ap^r Cb^r	This work
pMPO356	pMRB1-derived vector with the transcriptional fusion of P_{crcZ}::*gfpmut3-lacZ* (coord. -298 and -1 from TSS) cloned as *Eco*RI/*Bam*HI. Ap^r Cb^r	This work
pMPO357	pMRB1-derived vector with the transcriptional fusion of P_{crcY}::*gfpmut3-lacZ* (coord. -195 and -2 from TSS) cloned as *Eco*RI/*Bam*HI. Ap^r Cb^r	This work
pMPO358	pME6182-derivative expressing a truncated CbrA lacking the transmembrane domains ($CbrA^{\Delta28\text{-}1575}$) from the *lacI*^{q}-$P_{tac}$ expression system. Ap^r Gm^r	This work
pMPO420	Transcriptional fusion of P_{PP2810}::*lacZ* (coord. -472 to -10 from ATG) cloned as *Eco*RI/*Bam*HI into plasmid pMPO234. Ap^r Cb^r	[11]
pMPO434	pMPO1317-derived vector with a point mutation in the first ATG of *cbrX*. ATG to GCA (Met->Ala). Ap^r Gm^r	This work
pMPO483	pME6182-derived vector expressing a truncated ΔTM CbrA ($CbrA^{\Delta28\text{-}1575}$) from the P_{sal} promoter. Ap^r Gm^r	Unpublished
pMPO484	pEX18Tc -derived vector containing the *cbrA* flanking regions. Tc^r	This work

pMPO485	pMPO484-derived vector with a FRT-Km^{r}-FRT cassette between *cbrA* flanking regions. Tc^{r}, Km^{r}	This work
pMPO1259	pMPO1370-derived vector containing the *cbrX* sequence from pMPO1369. Ap^{r} Cb^{r}	This work
pMPO1261	*cbrA*'-'*gfpmut3* protein fusion expressed from its own promoter into pMPO1350. Ap^{r} Gm^{r}	This work
pMPO1314	Transcriptional fusion of P_{crcY}::*lacZ* (coord. -195 and -2 from TSS) cloned as *Eco*RI/*Bam*HI into plasmid pMPO234. Ap^{r} Cb^{r}	[12]
pMPO1316	Transcriptional fusion of P_{crcZ}::*lacZ* (coord. -298 and -1 from TSS) in cloned as *Eco*RI/*Bam*HI into plasmid pMPO234. Ap^{r} Cb^{r}	[12]
pMPO1317	*cbrA* gene from *P. putida* KT2442 in cloned as *Hind*III/*Sma*I into pME6182. Gm^{r} Ap^{r}	[4]
pMPO1324	pME6182-derived vector expressing a truncated CbrA lacking the PAS domain (CbrA$^{\Delta1881\text{-}2108}$) from its own promoter. Ap^{r} Gm^{r}	This work
pMPO1325	pME6182-derived vector expressing a truncated CbrA lacking the transmembrane domains (CbrA$^{\Delta4\text{-}1575}$) from its own promoter. Gm^{r} Ap^{r}	This work
pMPO1338	pMPO1324-derived vector with a null *Nco*I restriction site downstream the Multi Cloning Site. Ap^{r} Gm^{r}	This work
pMPO1344	pMPO1317-derived vector with the *cbrX* sequence bearing point mutation in the second predicted translational star site from ATG to GAT (Met>Asp). Gm^{r} Ap^{r}	This work
pMPO1347	pMPO1338-derived plasmid with a synthetic Shine-Dalgarno provided from RBS calculator into *cbrA* translational start.	This work
pMPO1348	pME6182-derived plasmid a ΔPAS deletion of CbrA (CbrA$^{\Delta1881\text{-}2108}$) expressed from *lacI*^{q}-P_{tac}. Gm^{r} Ap^{r}	This work
pMPO1349	pMPO1317-derived plasmid with ATG1&2 mutations at the *cbrX* coding sequence. Gm^{r} Ap^{r}	This work
pMPO1350	*gfpmut3* C-terminal protein fusion vector based on pUCSfiminiTn7BB-Gm with a modified MCS. Ap^{r} Gm^{r}	This work
pMPO1353	pMPO1350-derived vector containing the *lacI*^{q}-P_{tac} cassette from pIZ1016. Ap^{r} Gm^{r}	This work
pMPO1358	*cbrA*ΔTM'-'*gfpmut3* protein fusion into pMPO1353. Ap^{r} Gm^{r}	This work

pMPO1359	*cbrA'-'gfpmut3* protein fusion into pMPO1353. Ap^r Gm^r	This work
pMPO1367	*cbrA*ΔTM'-'*gfpmut3* protein fusion expressed from its own promoter into pMPO1350. Ap^r Gm^r	This work
pMPO1368	pMPO1317- derived plasmid with a ΔT deletion at the +3 *cbrX* coding sequence. Ap^r Gm^r	This work
pMPO1369	pMPO1317-derived plasmid with a ΔT deletion and a +C insertion at the *cbrX* coding sequence. Ap^r Gm^r	This work
pMPO1370	*cbrA'-'lacZ* translational fusion in pMPO200 cloned as *Eco*RI/*Xma*I carrying the sequence between positions -430 to +249 from ATG. Ap^r Cb^r	This work
pMPO1371	pMPO1370-derived vector containing the *cbrX* sequence from pMPO434 (ATG to GCA). Ap^r Cb^r	This work
pMPO1372	pMPO1370-derived vector containing the *cbrX* sequence from pMPO1344 (ATG to GAT). Ap^r Cb^r	This work
pMPO1373	pMPO1370-derived vector containing the *cbrX* sequence from pMPO1349 (ATG1 to GCA and ATG2 to GAT). Ap^r Cb^r	This work
pMPO1374	pMPO1370-derived vector containing the *cbrX* sequence from pMPO1368. Ap^r Cb^r	This work
pMRB1	pMPO234-derived broad-host range *gfpmut3-lacZ* transcriptional fusion vector. Ap^r	[10]
pRK2013	Helper plasmid for triparental mating. ColE1 replicon. Km^r	[13]
pUC18Sfi-miniTn7BB-Gm	pUC18Sfi-based delivery plasmid for the synthetic minitransposon miniTn7BB-Gm. Ap^r Gm^r	[10]
pUX-BF13	mini-Tn7 transposition helper plasmid. R6K replicon.Ap^r	[14]

Table S2. Oligonucleotides used in this study

Oligonucleotide	Sequence (5' to 3')
ATGpep_fwd	CGAGGCCAGC**GCA**TACATCTATC
ATGpep_rev	GATAGATGTA**TGC**GCTGGCCTCG
ATG2pep_fwd	CCCGGCCATC**GAT**GACTGGTGGA
ATG2pep_rev	TCCACCAGTC**ATC**GATGGCCGGG
ATG1-Tpep_fwd	AGGCCAGC**ATG**ACATCTATCGT
ATG1-Tpep_rev	ACGATAGATGT**CAT**GCTGGCCT
CbrA2103fwd	AAAGAGCTCCTGGCGCTGGACGGCCA
CbrA3EcoRev	ACCGAATTCCATCTCGTTGGCTCTGCA
CbrAcomplF	CCCAAGCTTAGAGGCTGATTTGCGCT
CbrAcomplR	CCCAATATTAGCGGATGATGGTTTCG
CbrAEcoRI_fwd	CAGGAATTCGCCACCCAGC
CbrAEcoRI_rev	GGCGAATTCCTGCGGCGAGGC
CbrAL1_fwd	ATAGAATTCGATCTACCGCTTTACCGACCG
CbrAL1_rev	ATAGGATCCCAGCGTTCGCCAAGGGTTTG
CbrAR_fwd	TATGGATCCGCCAGATCCTTGACCAGACC
CbrAR_rev	TTAAAGCTTCCACGTTGCCGTTCGATATC
CbrAsol_rev	TATGCATGCTCAATTCTCTCGACGGTC
CbrAsol1_fwd	ATAAAGCTTAAATAGAGAATAGAACCAAATAAGGAGGTCCCATGC CGATGAGCTTTAG
CbrATM-NcoI_rev	TATCCATGGGCCTTCCTGCAGTGGGGT
CbrATMSD_fwd	ATAAAGCTTAAATTACCAAACAGACACCCGCGGAAAAACAAAATG CCGATGAGCTTTAG
cbrB23 1Q	AAGACGAAACCATCATCCGC
cbrB74 2Q	TGGTTCCGCTCAAGCAGG
GFPfusion_fwd	ATAACTAGTCCCGGGATCCAAGCTTAGAGCTCGAGAGTAAAGGAGA AGAACTTTTC
PcbrAlongSmaI_rev	TATCCCGGGCAGGAATGCGCCAGACACCC
Pep3+C_fwd	TACCTGCTCTGGC**C**TGATCCTGATCG
Pep3+C_rev	CGATCAGGATCA**G**GCCAGAGCAGGTA
PstI_gfp_rev	AAAAAACTGCAGATTATTTTATTTGTATAGTTCATCCATGC
RT-CbrA_fwd	GTACCTCATGGTGCTGTTCG
RT-CbrA_rev	TAACAGGCAAGGAAGCCGTA
RT-cbrX_fwd	ATGTACATCTATCGTTTGGTCC
RT-cbrX_rev	CTAAAGCTCATCGGCATCTCGTT
RT-PcbrXA_fwd2	AGCCCGTAGCGCCCAACC

REFERENCES for supplementary Tables

1 Hanahan, D. Studies on transformation of Escherichia coli with plasmids. *J Mol Biol* **166**, 557-580 (1983).

2 Franklin, F. C., Bagdasarian, M., Bagdasarian, M. M. & Timmis, K. N. Molecular and functional analysis of the TOL plasmid pWWO from Pseudomonas putida and cloning of genes for the entire regulated aromatic ring meta cleavage pathway. *Proc Natl Acad Sci U S A* **78**, 7458-7462 (1981).

3 Ruiz-Manzano, A., Yuste, L. & Rojo, F. Levels and activity of the Pseudomonas putida global regulatory protein Crc vary according to growth conditions. *J Bacteriol* **187**, 3678-3686, doi:187/11/3678 [pii]10.1128/JB.187.11.3678-3686.2005 (2005).

4 Valentini, M. *et al.* Hierarchical management of carbon sources is regulated similarly by the CbrA/B systems in Pseudomonas aeruginosa and Pseudomonas putida. *Microbiology* **160**, 2243-2252, doi:10.1099/mic.0.078873-0 (2014).

5 Hoang, T. T., Karkhoff-Schweizer, R. R., Kutchma, A. J. & Schweizer, H. P. A broad-host-range Flp-FRT recombination system for site-specific excision of chromosomally-located DNA sequences: application for isolation of unmarked Pseudomonas aeruginosa mutants. *Gene* **212**, 77-86 (1998).

6 Martinez-Perez, O., Moreno-Ruiz, E., Floriano, B. & Santero, E. Regulation of tetralin biodegradation and identification of genes essential for expression of thn operons. *J Bacteriol* **186**, 6101-6109, doi:10.1128/JB.186.18.6101-6109.2004186/18/6101 [pii] (2004).

7 Humair, B., Gonzalez, N., Mossialos, D., Reimmann, C. & Haas, D. Temperature-responsive sensing regulates biocontrol factor expression in Pseudomonas fluorescens CHA0. *ISME J* **3**, 955-965, doi:10.1038/ismej.2009.42 (2009).

8 Garcia-Gonzalez, V., Govantes, F., Porrua, O. & Santero, E. Regulation of the Pseudomonas sp. strain ADP cyanuric acid degradation operon. *J Bacteriol* **187**, 155-167, doi:10.1128/JB.187.1.155-167.2005 (2005).

9 Porrua, O., Garcia-Gonzalez, V., Santero, E., Shingler, V. & Govantes, F. Activation and repression of a sigmaN-dependent promoter naturally lacking upstream activation sequences. *Mol Microbiol* **73**, 419-433, doi:10.1111/j.1365-2958.2009.06779.x (2009).

10 Jimenez-Fernandez, A., Lopez-Sanchez, A., Calero, P. & Govantes, F. The c-di-GMP phosphodiesterase BifA regulates biofilm development in Pseudomonas putida. *Environ Microbiol Rep* **7**, 78-84 (2015).

11 Barroso, R. *et al.* The CbrB Regulon: Promoter dissection reveals novel insights into the CbrAB expression network in Pseudomonas putida. *PLoS One* **13**, e0209191, doi:10.1371/journal.pone.0209191 (2018).

12 Garcia-Maurino, S. M., Perez-Martinez, I., Amador, C. I., Canosa, I. & Santero, E. Transcriptional activation of the CrcZ and CrcY regulatory RNAs by the CbrB response regulator in Pseudomonas putida. *Mol Microbiol* **89**, 189-205, doi:10.1111/mmi.12270 (2013).

13 Figurski, D. H. & Helinski, D. R. Replication of an origin-containing derivative of plasmid RK2 dependent on a plasmid function provided in trans. *Proc Natl Acad Sci U S A* **76**, 1648-1652 (1979).

14 Bao, Y., Lies, D. P., Fu, H. & Roberts, G. P. An improved Tn7-based system for the single-copy insertion of cloned genes into chromosomes of gram-negative bacteria. *Gene* **109**, 167-168 (1991).
